# Quantifying the Effect of Polymer Blending through Molecular Modelling of Cyanurate Polymers

**DOI:** 10.1371/journal.pone.0044487

**Published:** 2012-09-06

**Authors:** Alasdair O. Crawford, Ian Hamerton, Gabriel Cavalli, Brendan J. Howlin

**Affiliations:** Department of Chemistry, University of Surrey, Guildford, Surrey, United Kingdom; University of Leeds, United Kingdom

## Abstract

Modification of polymer properties by blending is a common practice in the polymer industry. We report here a study of blends of cyanurate polymers by molecular modelling that shows that the final experimentally determined properties can be predicted from first principles modelling to a good degree of accuracy. There is always a compromise between simulation length, accuracy and speed of prediction. A comparison of simulation times shows that 125ps of molecular dynamics simulation at each temperature provides the optimum compromise for models of this size with current technology. This study opens up the possibility of computer aided design of polymer blends with desired physical and mechanical properties.

## Introduction

Cyanate esters constitute a family of addition cured high performance, thermosetting polymers, which occupy a niche intermediate between high glass transition temperature (T_g_), tetrafunctional epoxy resins and bismaleimides (BMIs) [1]. The combination of favourable thermal and mechanical performance (dry T_g_ values of 270–300°C are common with a strain at break of over 5–8%) coupled with low dielectric loss properties (a low dielectric constant of *ca.* 2.7 with a loss tangent of 0.003 is typical [1]) make cured cyanates attractive and able to offer a unique property profile. Although requiring toughening for some engineering applications, cyanates can be combined with inherently tough engineering thermoplastics (HexPly 954-2A, G_IC_ = 250 J/m^2^) [2] or elastomers (HexPly 953-3, G_IC_ = 450 J/m^2^) [3] to yield impressive enhancements. In this form they typically find application as matrices in advanced composites (either in combination with epoxy resins in aerospace applications [4]) or with BMIs as dielectric polymers in the microelectronics industry [5]. The rationale for the preparation of the binary blends presented here is to examine the potential for deriving improved properties (physical and mechanical) through the co-reaction of chemically compatible monomers. To date, aside from isolated studies [6], it appears that little systematic work has been carried out to examine the potential benefits of blending and co-curing polycyanate monomers. In the current study, we have examined three common cyanate species and the binary blends thereof by simulating selected properties using atomistic modelling in our drive to improve our predictive capability.

Molecular modelling of polymers is a growing area and it has been used in a wide variety of polymeric systems. By far the most effort has been concentrated on epoxies, owing to their general usefulness. Reports have predicted the structure, mechanical properties and moisture diffusion in epoxy resins [7], [8]–[13]. Other thermosetting polymers have also been modelled including, polycyanurates [14], polybenzoxazines [15], polyimides (in particular gas permeation across polyimide membranes) [16]–[18] and cyclohexanone formaldehyde resins (plastic printing) [19]. Non thermosetting polymers have included polyethylene oxides [20], polysiloxanes (glass transition temperature) [21], [22] and polyethylene terepthalate (gas diffusion) [23]. Recently the field has moved into the modelling of nanocomposites with carbon nanotube reinforced composites becoming of interest [24], [25].

It is fundamental to our approach that the simulations that are performed are always supported by empirical data, either single crystal data in the formation of structures or from physical or mechanical measurements when determining properties for the final polymer. In this paper we report a systematic study of the blending of the three cyanates by molecular simulation using the software suite Materials Studio [26]. This method is generally applicable to all crosslinked resin systems. There is always a compromise between how long the simulation takes and the accuracy of the predicted results. If it takes much longer to simulate the system than it takes to synthesize and measure the properties predicted then the method will be of limited usefulness in property prediction. This is a particular problem with synthetic polymers as there is an on-going debate on whether it is possible to equilibrate a long polymer chain by molecular mechanics, see for example [27].

## Methods

### Materials

**Figure 1:**
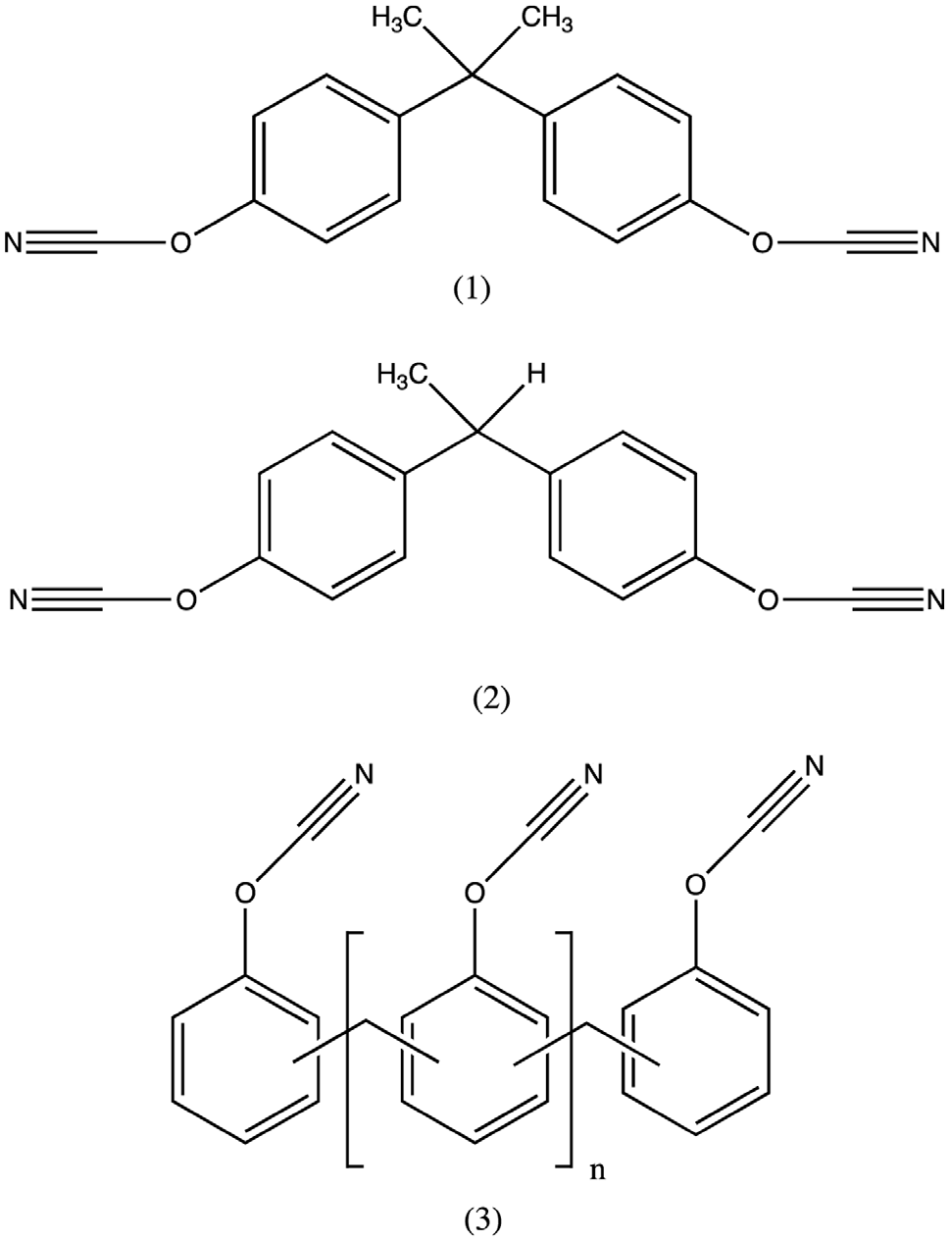
Cyanate ester structures studied in this work. (*N.B.*, in this instance n = 1 in structure (3)).

**Figure 2:**
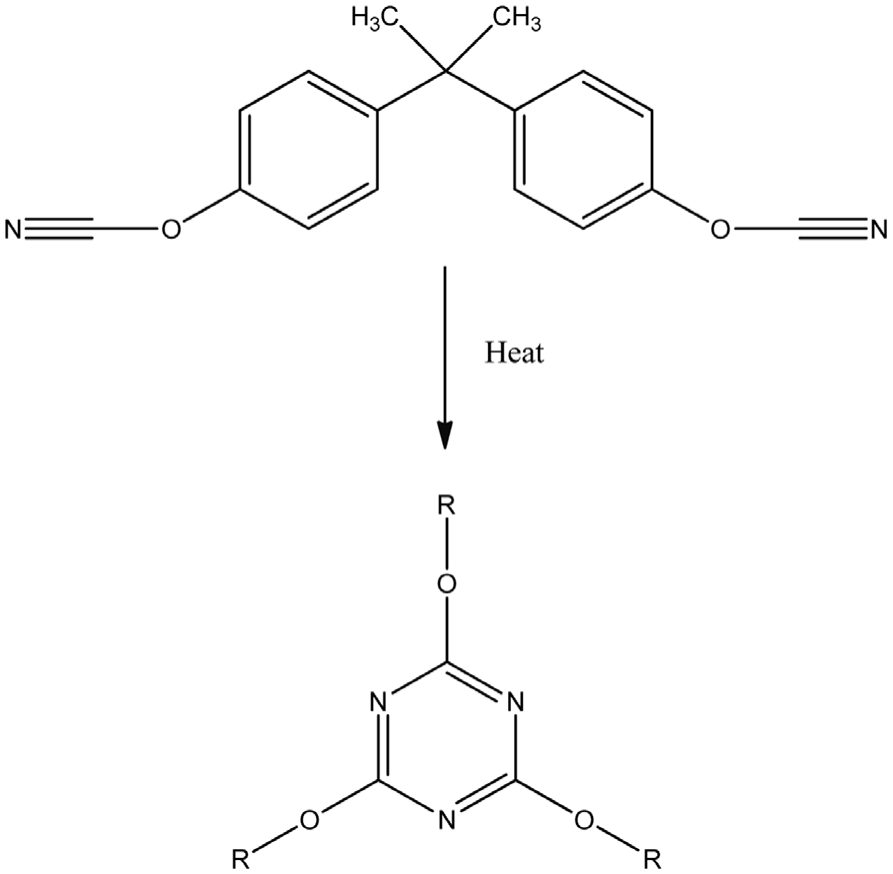
Scheme of cyclotrimerisation to triazine.

The dicyanate ester monomers: 2,2-*bis*(4-cyanatophenyl)propane (1), hereafter termed BADCy 1,1-*bis*(4-dicyanatophenyl)ethane (2) (LECy), and the oligomeric phenolic cyanate (average value of n = 1) (3) (PT30) were the three monomers simulated in this work. (Figure 1). Experimental values of the materials were also obtained by the methods described in Crawford et. al, 2012 [28]. The blends simulated are presented in Table 1. The monomers polymerise by cyclotrimerisation, where three cyanate groups combine to form a six membered cyanurate ring, so the resulting polymer is a cross linked network composed of cyanurate rings joined by the bridging groups attached to the cyanate moieties (Figure 2). *N.B.* cured blends are denoted by the use of square brackets to differentiate them from the corresponding monomer blend *i.e.* monomer blend (1_90_–2_10_) becomes polycyanurate [1_90_–2_10_] following cure.

**Table 1:** Designation of monomers and blends examined in this work. The data represent the percentage of each monomer in the blend.

Sample Designation	(1)	(2)	(3)
(1)	100	0	0
(2)	0	100	0
(3)	0	0	100
(1_50_–2_50_)	50	50	0
(1_60_–2_40_)	60	40	0
(1_70_–2_30_)	70	30	0
(1_80_–2_20_)	80	20	0
(1_90_–2_10_)	90	10	0
(3_50_–1_50_)	50	0	50
(3_60_–1_40_)	40	0	60
(3_70_–1_30_)	30	0	70
(3_80_–1_20_)	20	0	80
(3_90_–1_10_)	10	0	90
(3_50_–2_50_)	0	50	50
(3_60_–2_40_)	0	40	60
(3_70_–2_30_)	0	30	70
(3_80_–2_20_)	0	20	80
(3_90_–2_10_)	0	10	90

### Simulation

The Materials Studio molecular modelling suite (Accelrys Inc.) was utilised in this work [26] using in house PCs (*e.g.* a Dell PowerEdge 1950, 2×Quad Core Intel Xeon E5140 2.33 GHz, 8 GB RAM, 500 GB HDD). The Discover module was used for general simulation requirements, such as geometry optimisation and molecular dynamics as well as molecular mechanical analysis to predict values for tensile, bulk modulus, shear modulus, Poisson’s ratio and the Lamé constants. The Amorphous Cell module was used to build amorphous, homogenous 3D cells composed of molecules that were drawn *in silico*. It also has a number of protocols designed to make greater use of the Discover module, of specific interest is the temperature cycling protocol, which can be used for T_g_ prediction. All simulations were performed in the bulk state, i.e. without the addition of solvent as there is no added solvent in epoxy resin cure. Potential energies were calculated using the Polymer Consistent Force Field (PCFF) [29]. An attempt was made to use the COMPASS [30] forcefield but this implementation does not cope with the cyanate group. The *Legacy* module within Materials Studio was used to construct an amorphous cell. The amorphous cell contained an average of 4480 atoms. The simulations were also run with cell sizes double, thrice and four times this number. A unit cell of the appropriate volume was packed randomly with the correct number of monomers, either as single monomers as in the neat resin work or in the correct mole fraction in the case of the blends. The cyanate groups were inspected and those within 4.5 Å of each other were bonded manually to form 1,3,5-triazine rings and a minimization of 5000 iterations was carried out to relieve the strain using the *Discover* minimization module to a convergence of 1000 kcal mol^−1^ Å^−1^. A *Conjugate Gradient* algorithm [31] was carried out to a convergence of 100 kcal mol^−^ Å^−1^. As all monomers were assumed to be equally coreactive the nature of the final structure depended ultimately on the initial random arrangement of the monomers. Hence it was possible to generate homogenous networks or blocked semi-interpenetrating networks depending on the starting conditions. Bonds were reacted through the faces of the unit cell to neighbouring periodic boxes to simulate an infinite three-dimensional highly cross-linked network. After the final cyclotrimerization the system was submitted to a final minimization of 10,000 iterations. The NPT ensemble with a time step of 1 fs was utilized with the Andersen thermostat [32] at a Pressure of 0.1 MPa under the Parrinello Barostat [33]. PCFF was used with the atomic Van der Waals summation, a cut-off of 10.00 Å, a spline width of 3.00 Å and a buffer width of 1.00 Å. 51 MD simulations were run between 773 K (500°C) and 273 K (0°C) in decrements of 10 K; at each temperature stage a 125 ps MD simulation was carried out. In accord with the problem of equilibrating long polymer chains, the simulations were also run for 1 ns at each temperature stage. A plot of calculated cell density was plotted against simulation temperature to determine both the T_g_ and the degradation onset temperature.

**Figure 3:**
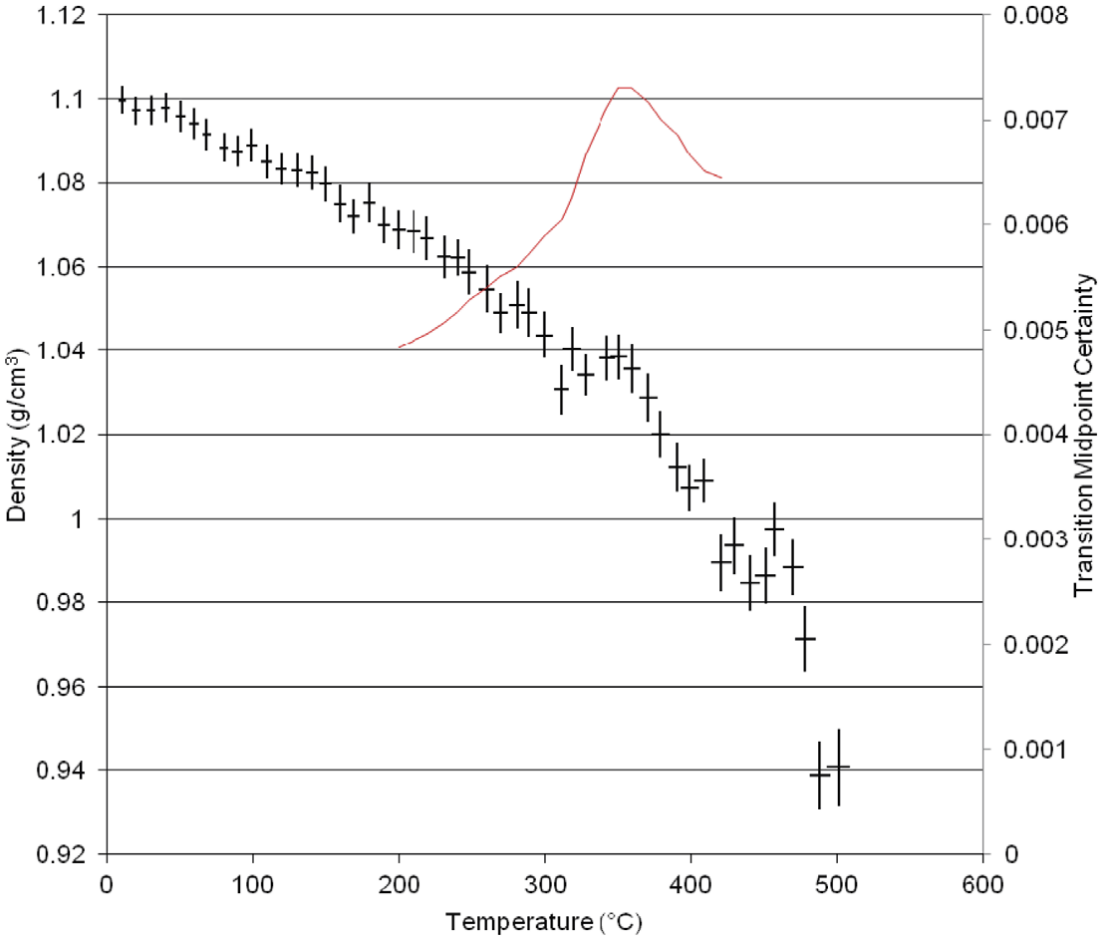
Results of Molecular dynamics simulation of density versus temperature for the homopolymer of *BADCy.*

## Results and Discussion

### Effect of Model Size

Increasing the model size had the effect of improving the statistics of the runs but made the T_g_ value more difficult to determine. In essence we are providing more degrees of freedom for the system with the larger models and thereby increasing the error (by comparison with experiment). The equilibration problem increases with larger model sizes. A survey of the data produced by varying cell size is given in Table S1.

**Figure 4:**
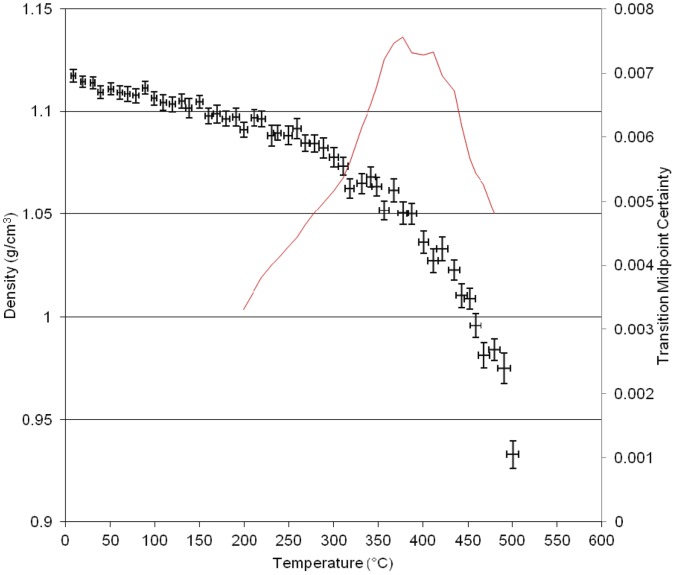
Results of molecular dynamics simulation of density versus temperature for the homopolymer of *LECy*.

**Figure 5:**
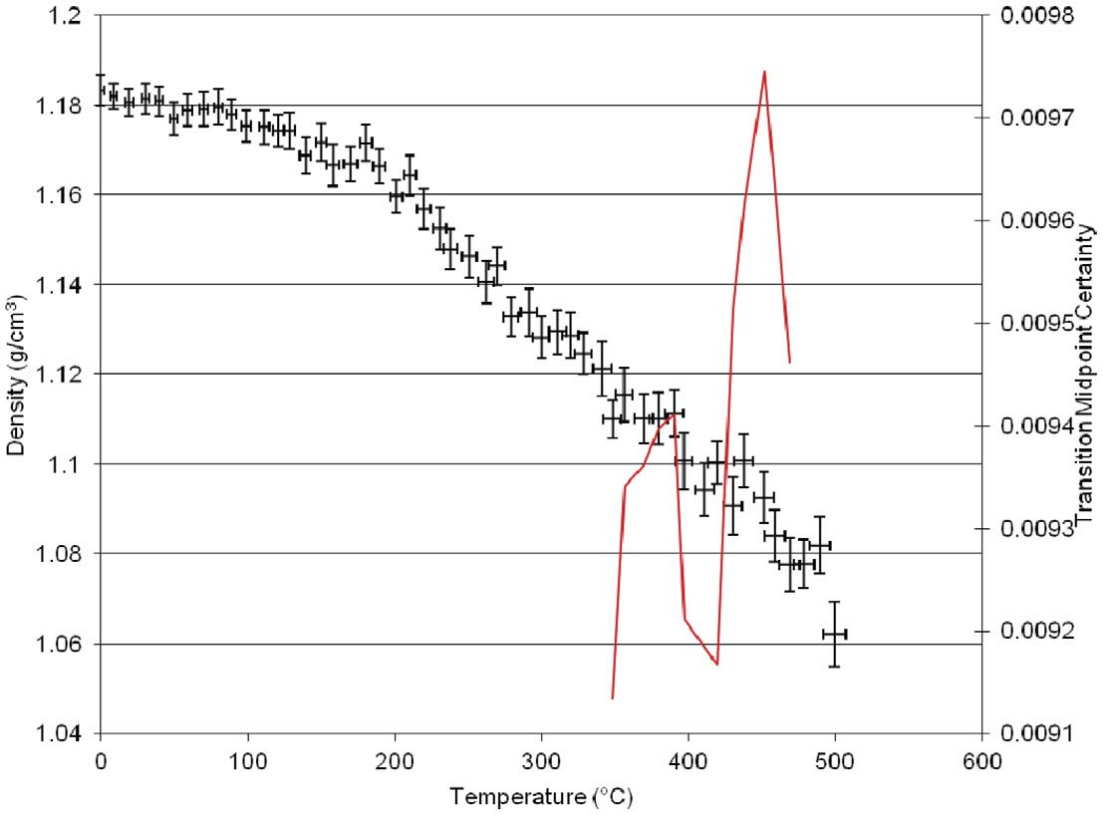
Results of molecular dynamics simulation of density versus temperature for the homopolymer of PT30.

### Effect of Simulation Length

The simulations that were run for 1 ns showed that, the statistics (in terms of the scatter of points) were much improved. However, the determination of the Tg value was more problematic, with a shift in the predicted value compared to the experiment. Additionally, the longer timescale served to remove the high temperature transition, which we have assigned to the thermal degradation of the material.

### Hysteresis of the Model

It has been noticed in previous work by ourselves and others that in order to get results that correlate with experiment, it is necessary to run the simulation by cooling down from the high temperature regime to the lower. If the simulation is run by heating up the system, the T_g_ value determined can vary by 30 degrees. This hysteresis is a function of the equilibration problem mentioned above and the cooling down is thought to serve to freeze out high energy motions thereby providing a better correlation with experiment.

**Figure 6:**
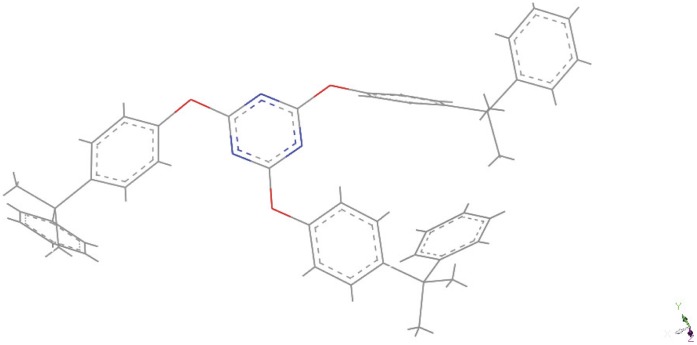
Image of delocalised triazine ring formed through polymerisation.

**Figure 7:**
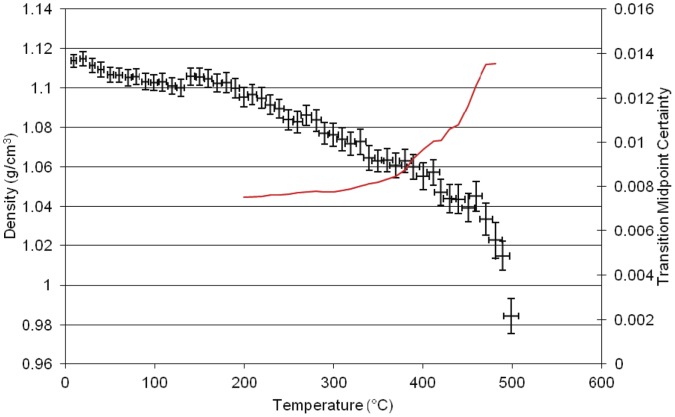
Results of molecular dynamics simulation of density versus temperature for the blend of monomer 1 and 3 in a 70∶30 ratio using alternating single and double bonds.

**Figure 8:**
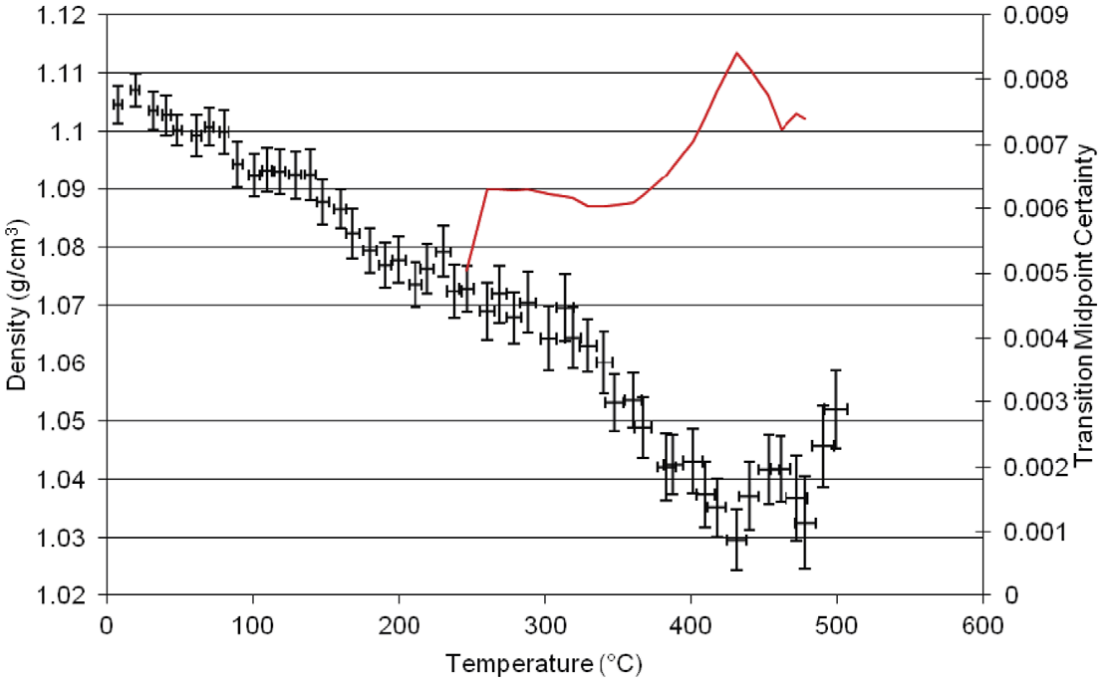
Results of molecular dynamics simulation of density versus temperature for the blend of monomer 1 and 3 in a 70∶30 ratio using aromatic bonds.

### Neat Polymers

The plot of simulated density versus temperature from the MD simulation for the neat polycyanurate [BADCy] is shown in Figure 3. The black crosses are the equilibrated volume at that simulated temperature and the red solid line is the derivative of the plot using the method outlined in Hall et al. [34]. There are several features apparent in the plot which correlate well with empirical data. At lower temperatures (80°C), a small drop in the density is apparent, which we have termed the γ transition. This transition corresponds to the melt endotherm in Differential Scanning Calorimetry data of the uncured monomer. We have observed this experimentally at 82°C [27]. A second drop is also observed at 175°C corresponding to the β transition observed in the DMTA data for this polymer [28]. The glass transition (α transition) is marked by the larger fall at 260°C. The large drop in density at 350°C and 420°C may correspond to the thermal degradation of the polymer. The group bridging the two aromatic rings in this polymer has two pendant methyl groups.

The simulation of the second neat polycyanurate [LECy] (Figure 4) shows a higher γ transition at 195°C, a higher β transition at 235°C and a higher α transition at 310°C with the main change in structure being the replacement of one of the pendant methyl groups in the bridging group by a hydrogen. This small change in atomic architecture has larger implications for the thermo- mechanical properties of the resulting polymer. The simulation of the third neat polycyanurate [PT30] (Figure 5)shows the γ transition at 90°C, the β transition at 260°C and the highest α transition (T_g_) at 360°C, with the Degradation (T_d_) at ca. 400°C and 480°C. The higher glass transition temperature results from the much reduced flexibility of this polymer with both of the pendant groups effectively reduced to hydrogens.

**Table 2:** Thermal data from molecular simulation (Transition and onset temperatures in °C).

Model Composition	No. of atoms	α Transition (T_g_)		β Transition	γ Transition	Degradation onset (T_d_)	
(1)	4778	260		175	80	350	420
(2)	5553	310		235	195	405	
(3)	5230	360		260	90	400	480
[(1)_50_–(2)_50_]	3580	295		220	80	390	440
[(1)_60_–(2)_40_]	4470	290		195	100	395	460
[(1)_70_–(2)_30_]	4604	250		350	110	400	430
[(1)_80_–(2)_20_]	4276	280		310	75	380	420
[(1)_90_–(2)_10_]	4380	295		220	80	400	440
[(3)_50_–(2)_50_]	5348	350		270	80	395	420
[(3)_60_–(2)_40_]	4704	350		250	110	405	420
[(3)_70_–(2)_30_]	5658	300	350	230	105	400	420
[(3)_80_–(2)_20_]	5920	370		260	120	410	460
[(3)_90_–(2)_10_]	5552	290	360	180	95	410	450
[(3)_50_–(1)_50_]	5409	305	360	175	75	400	450
[(3)_60_–(1)_40_]	5798	305	360	160	80	420	460
[(3)_70_–(1)_30_]	6016	280	370	205	80	480	
[(3)_80_–(1)_20_]	6224	260	360	110	75	400	450
[(3)_90_–(1)_10_]	6040	310	370	120	60	405	490

**Table 3:** Comparison of predicted and experimental thermal data (all +/−5°C).

Blend	Predicted T_g_(1/2)	Actual T_g_	Predicted T_d_(1/2)	Actual T_d_
[(1)_50_–(2)_50_]	295	291.83	390/440	414
[(1)_60_–(2)_40_]	290	291.59	395/460	411
[(1)_70_–(2)_30_]	250	292.72	400/430	416
[(1)_80_–(2)_20_]	280	286.3	380/420	413
[(1)_90_–(2)_10_]	295	287.76	400/440	400
[(3)_50_–(2)_50_]	350	336.9	395/420	420
[(3)_60_–(2)_40_]	350	331.83	405/420	420
[(3)_70_–(2)_30_]	300/350	331.72	400/420	408
[(3)_80_–(2)_20_]	370	325.27	410/460	420
[(3)_90_–(2)_10_]	290/360		410/450	418
[(3)_50_–(1)_50_]	305/360	311.82/343.83	400/450	416
[(3)_60_–(1)_40_]	305/360	307.82	420/460	419
[(3)_70_–(1)_30_]	280/370	380.62	480	420
[(3)_80_–(1)_20_]	260/360	325.27	400/450	420
[(3)_90_–(1)_10_]	310/370		405/490	419

### Effects of Parameterisation

In previous work we reparameterised the Dreiding force field [35] specifically for cyanurate rings [36]. A study of the available x-ray crystal structure data for cyanurate rings showed that the bond lengths are not equal and that the ring was slightly puckered. The new parameterization improved the simulation results significantly. The second generation force field used in this work is generally better all round than the first generation one used earlier but there is still the option to use alternating single and double bonds in the cyanurate rings or to use delocalized aromatic rings. By default Materials Studio chooses atom types as alternating single and double bonds but we tested the aromatic rings by running the simulations with wholly aromatic rings (Figure 6). An example is shown below for the 70∶30 blend of monomer 1 and 2 (Figures 7 and 8). There is more scatter on the data for the aromatic parameterization and the thermal events are different, i.e. α transition (Tg) = 250°C, β transition = 350°C, γ transition = 110°C, degradation (Td) = i) 400°C ii) 430°C for the alternating single and double bonds to α transition (Tg) = 295°C, β transition = 210°C, γ transition = 90°C, degradation (Td) = i) 420°C ii) 480°C for the aromatic parameterization. The experimentally determined values of the α transition (Tg) = 292°C and the degradation (Td) = i) 416°C. Hence the aromatic parameterization gives the better comparison with experiment, so was the one used.

**Figure 9:**
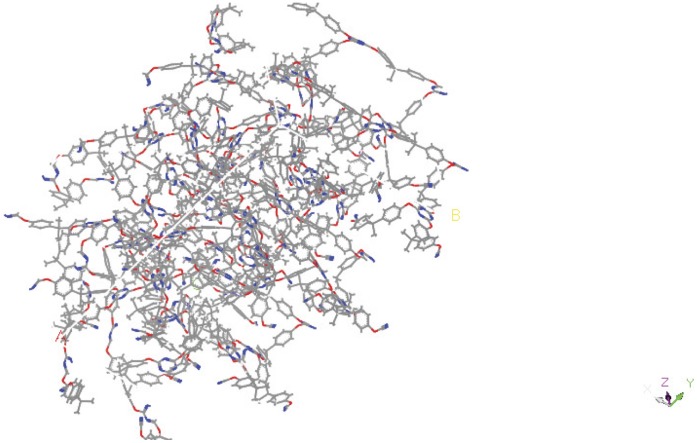
Image of [3_70_ –**1_30_].**

**Figure 10:**
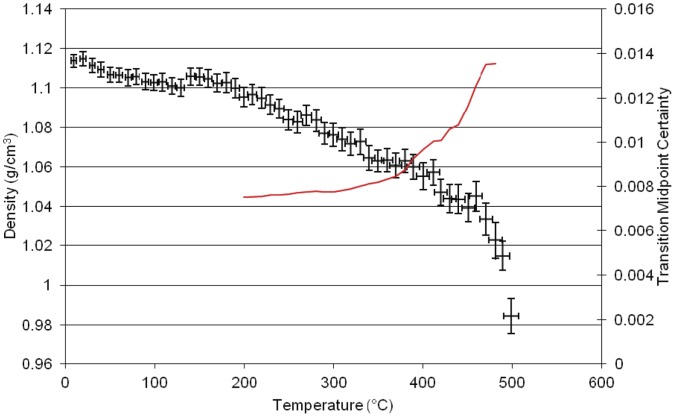
Results of molecular dynamics simulation of density versus temperature for the blend of *BADCy:LECy* in a 50∶50 blend.

### Binary Blends

The corresponding data for the binary blend (50% of the first two polycyanurates) comprises elements of both networks: displaying a flatter response at lower temperatures (where the behaviour of monomer 1 predominates evidenced by the lower γ transition at 80°C similar to the first monomer, but appears more similar to monomer 2 at higher temperature with α transition (T_g_) at 295°C and the β transition at 220°C. Interestingly the blending has pushed the degradation onset to 390°C with the second stage at 440°C, which is more similar to the third monomer.

A systematic study of the blends of the three monomers was carried out from 50∶50 of each of the monomers to 90∶10 and the alpha, beta, gamma and degradation onset temperatures for each were simulated. These results are presented in Table 2. It can be seen that there is a general trend towards the thermal data peaking for the 80∶20 and 90∶10 blends, although this is not clear cut in all cases. The predicted alpha transitions and degradation onset temperatures were compared with actual experimental values for all cases (Table 3). A visual comparison of the experimental TGA and DMTA data plotted against the simulated data is given in supporting information, Table S2. As noticed for the binary blend discussed earlier there is a general trend to favour the behaviour of the less thermally stable monomer at lower temperatures and the more thermally stable monomer at higher temperatures. There is good agreement in almost all cases except for the blend of 70∶30 of monomer 3 and 1, where there are 2 inflexions in the simulated data at 280 and 370 K and the experimental data gives a value of 380 K. The three dimensional structure of this blend is shown in Figure 9. This blend also shows an anomaly at the 50∶50 blend, again of monomer 3 and 1 (Figure 10). Here there are also 2 inflexions but also 2 inflexions in the experimental data, which are close to the experimental values. This blend seems to be behaving as 2 independent networks both in the simulation and also in the experiment.

Molecular simulation of cyanurate polymers is shown to reproduce with a good degree of accuracy the thermal events occurring experimentally for three different cyanate containing monomers. Furthermore it is also possible to simulate the thermal events of binary blends of the three monomers, again with good agreement to experiment. These thermal events, particularly the α transition, more commonly known as the Tg, are important in selecting materials for end use applications. The Tg determines the thermal softening point of the polymer and is crucially important in e.g. aerospace applications where it is not desirable for the polymer to exceed its Tg in use. Therefore molecular simulation can be used as a tool to screen and design polymers and blends thereof for end use applications.

## Supporting Information

Table S1 **A survey of the data produced by varying cell size.**
(DOCX)

Table S2 **A visual comparison of the experimental TGA and DMTA data plotted against the simulated data.**
(DOCX)
