# Supplementary material for: Quantifying the Effect of Polymer Blending through Molecular Modelling of Cyanurate Polymers
Source: PLoS One. 2012 Sep 6;7(9):e44487. doi: 10.1371/journal.pone.0044487 (PMC3435312; doi:10.1371/journal.pone.0044487)
Supplement: Table S1 — A survey of the data produced by varying cell size. (DOCX) [file pone.0044487.s001.docx]

**Size comparisons between models**

BADCy:LECy (50:50) [1_50_-2_50_] (delocalised triazine rings) **FIGURE 9 IN PAPER**


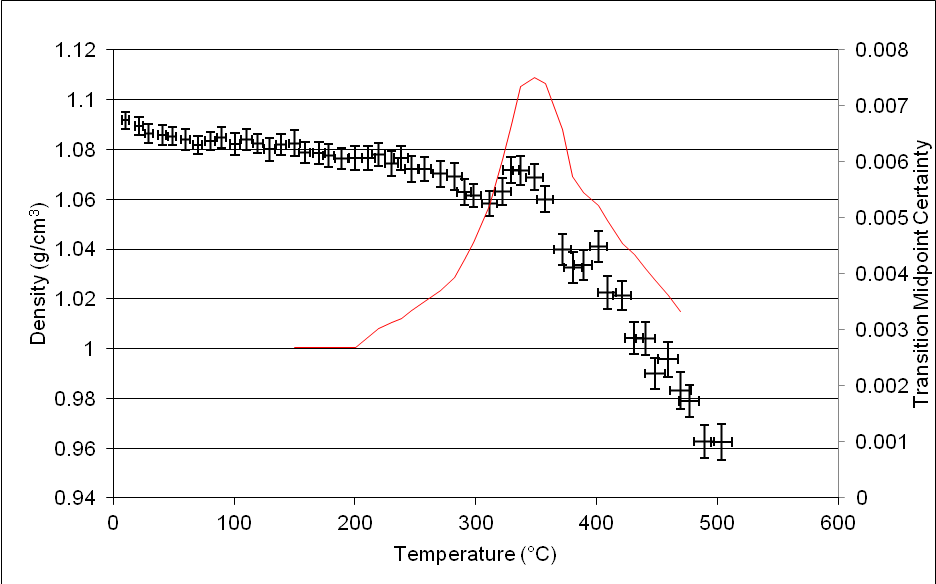


Model size: 3217 atoms

BADCy:LECy (50:50) [1_50_-2_50_] (delocalised triazine rings, larger model)


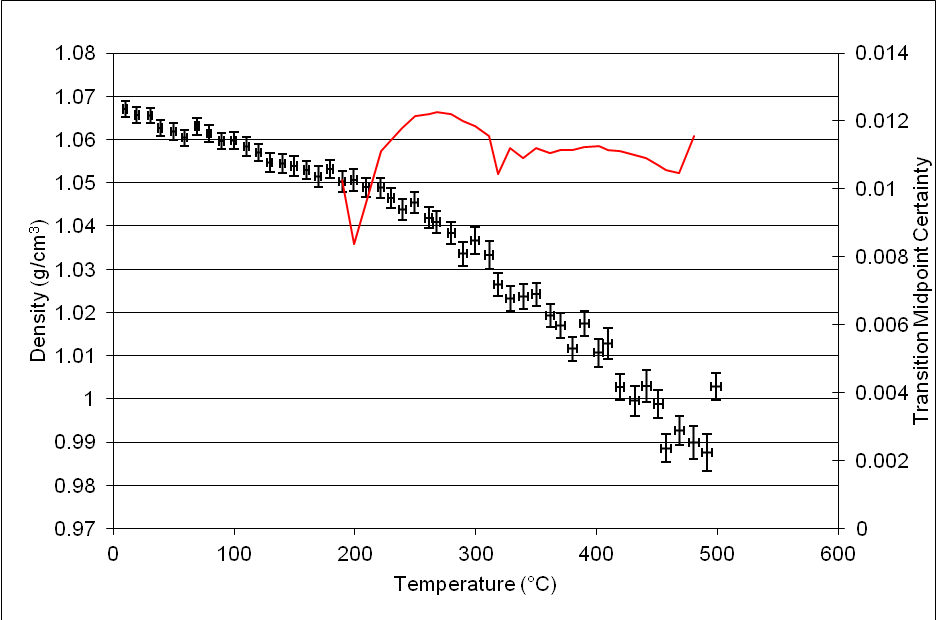


Model size: 12868 atoms

BADCy:LECy (70:30) [1_70_-2_30_] (alternating single, double bonds) **FIGURE 7 IN PAPER**


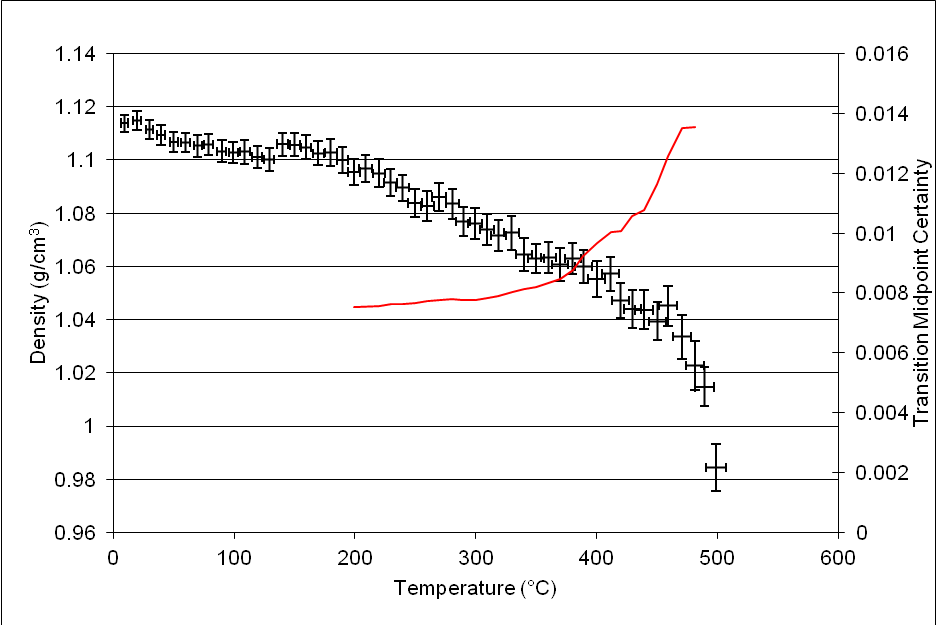


Model size: 4092 atoms

BADCy:LECy (70:30) [1_70_-2_30_] (delocalised triazines) **FIGURE 8 IN PAPER**


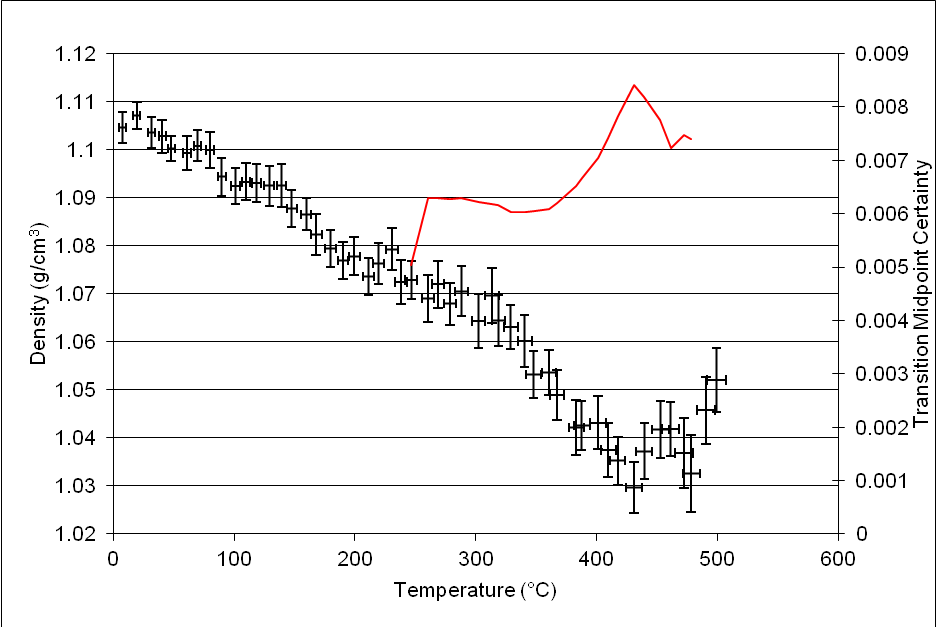


Model size: 4092 atoms

BADCy:LECy (70:30) [1_70_-2_30_] (larger model with delocalised triazines)


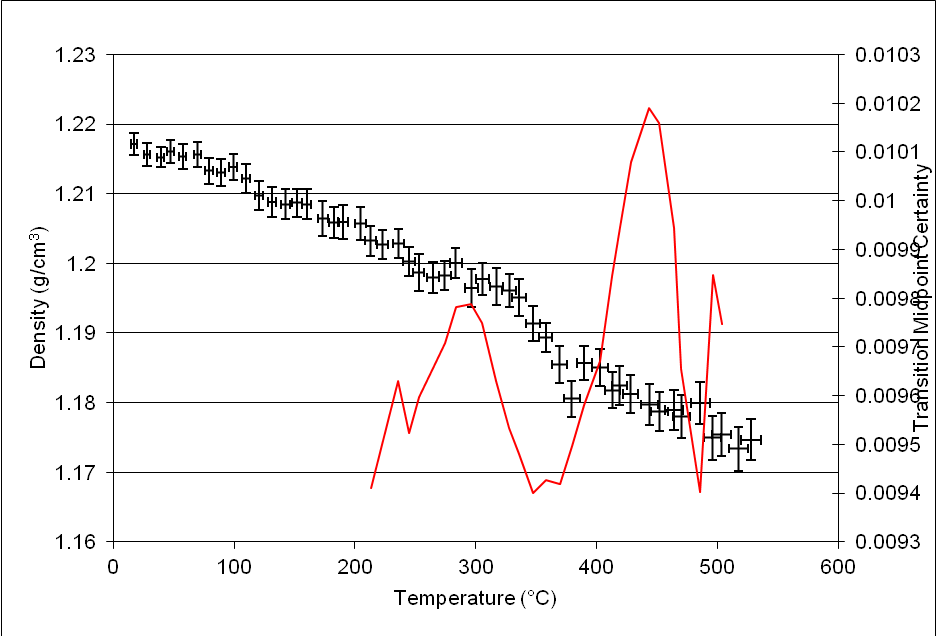


Model size: 8184 atoms

**Effect of increased dynamics time (100ps 🡪 1ns)**

PT30:BADCy (60:40) [3_60_-1_40_] (delocalised triazines)


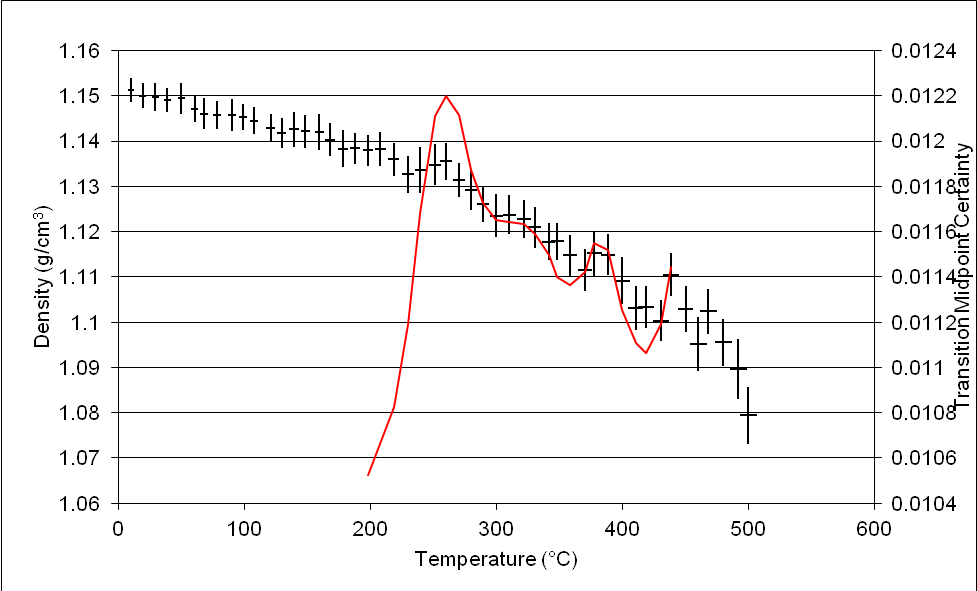


Model size: 5650 atoms

PT30:BADCy (60:40) [3_60_-1_40_] (larger model with delocalised triazines)


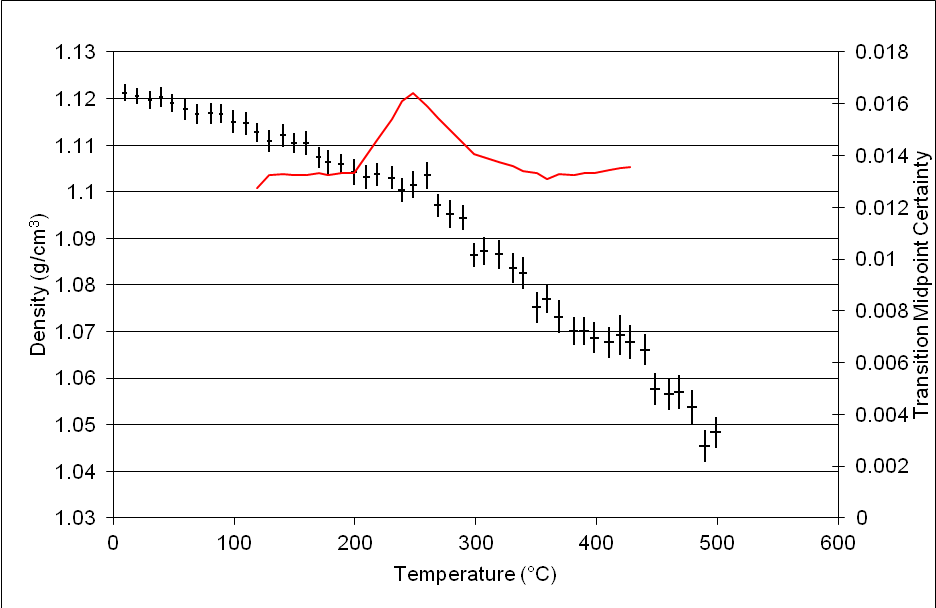


Model size: 11286 atoms

PT30:BADCy (60:40) [3_60_-1_40_] (larger model with delocalised triazines and dynamics time of 1ns)


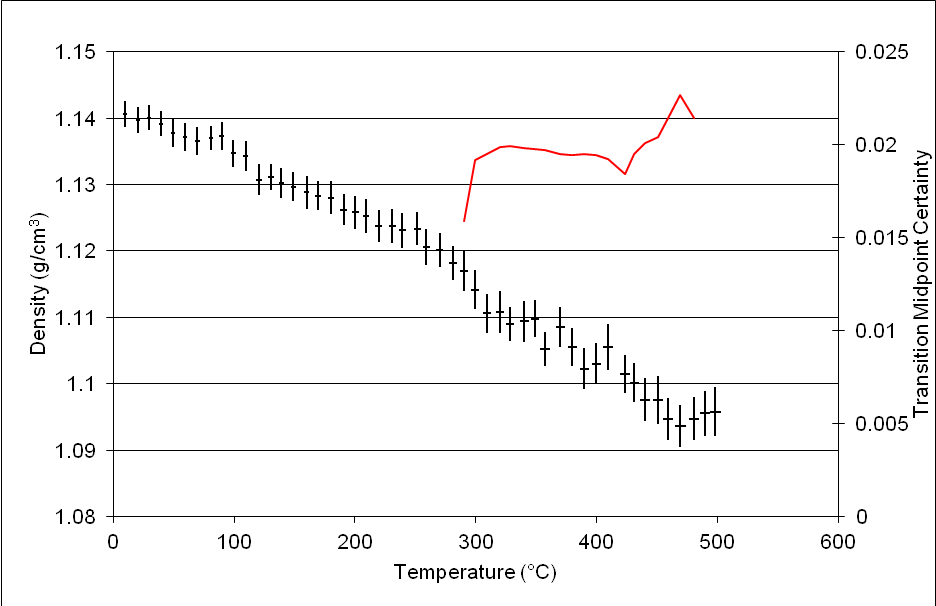


Model size: 11286 atoms

BADCy:LECy (70:30) [1_70_-2_30_] (alternating single and double bonds)


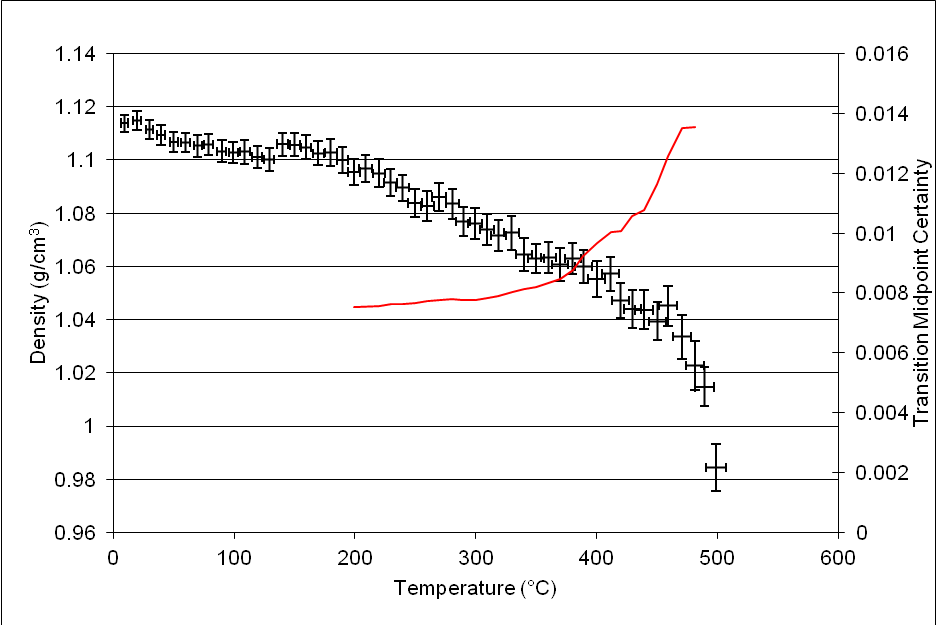


Model size: 4092 atoms

BADCy:LECy (70:30) [1_70_-2_30_] (larger model with delocalised triazines)


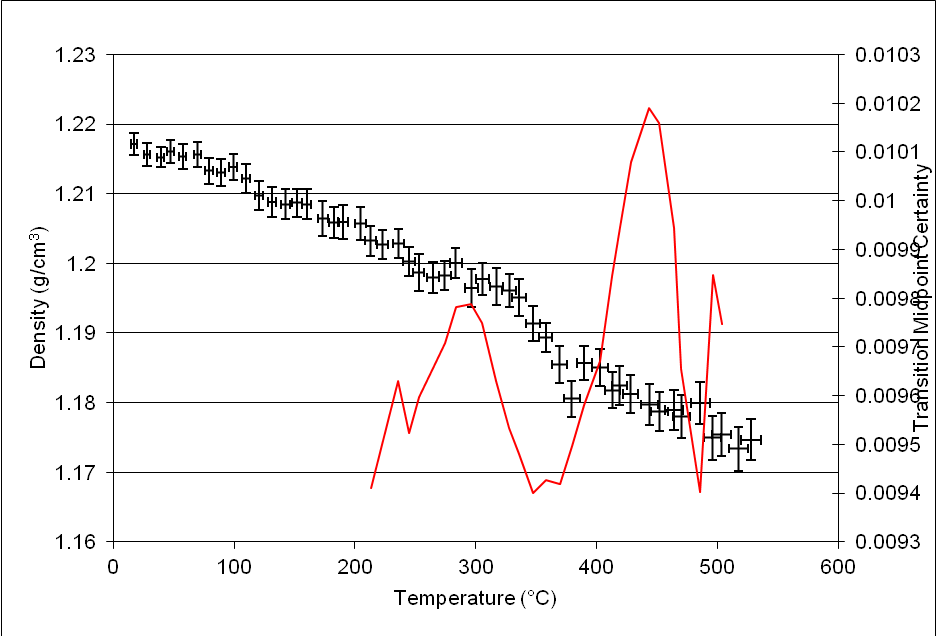


Model size: 8184 atoms

BADCy:LECy (70:30) [1_70_-2_30_] (larger model with delocalised triazines and dynamics time of 1ns)


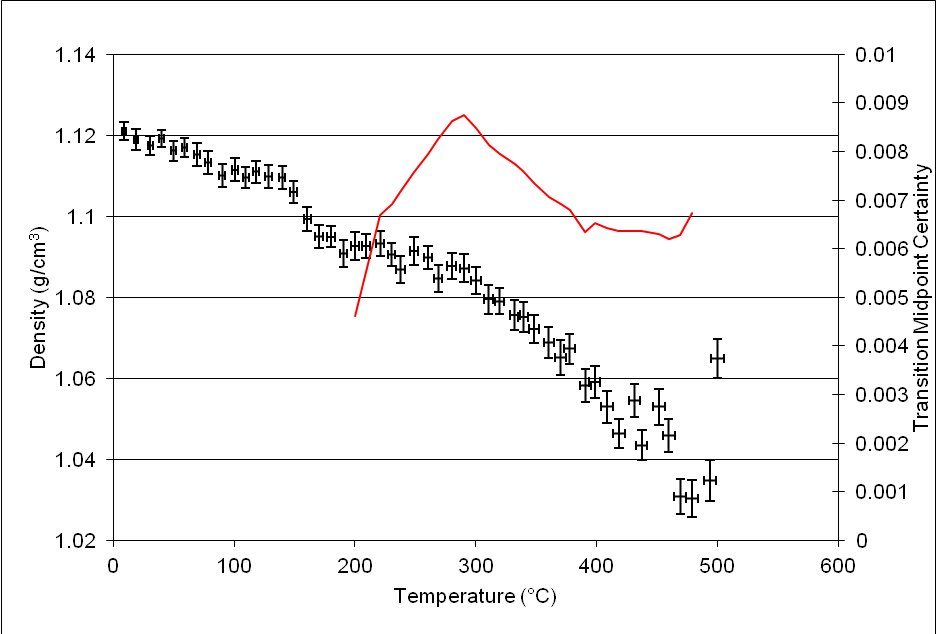


Model size: 8184 atoms

BADCy:LECy (70:30) [1_70_-2_30_] (larger model with delocalised triazines, dynamics time of 1ns and temp cycle run in reverse)


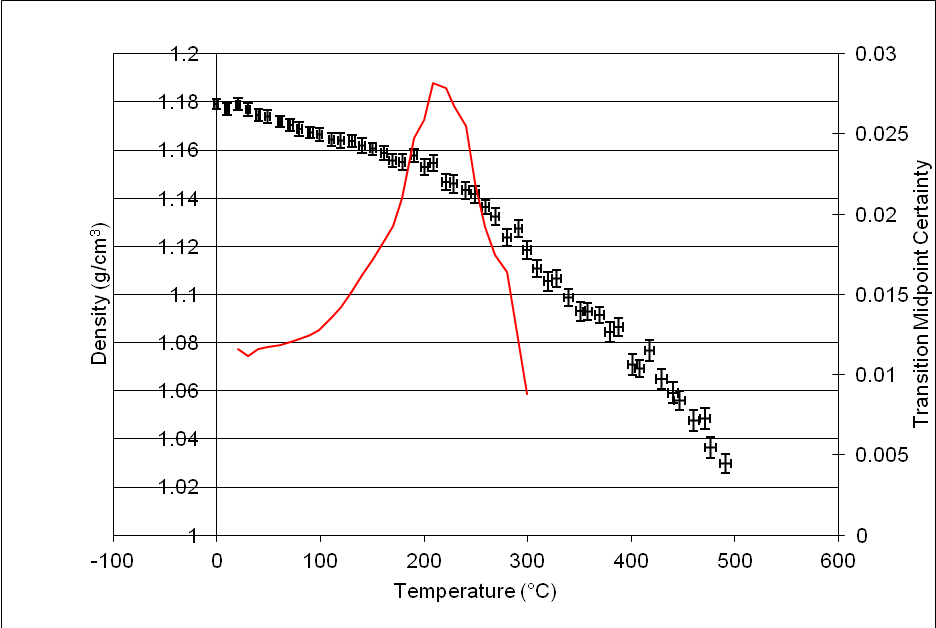


Model size: 8184 atoms
