# Supplementary material for: Quantifying the Effect of Polymer Blending through Molecular Modelling of Cyanurate Polymers
Source: PLoS One. 2012 Sep 6;7(9):e44487. doi: 10.1371/journal.pone.0044487 (PMC3435312; doi:10.1371/journal.pone.0044487)

Comparison between modelled and actual data of binary CE blends

**1**_50_-**2**_50_

TGA


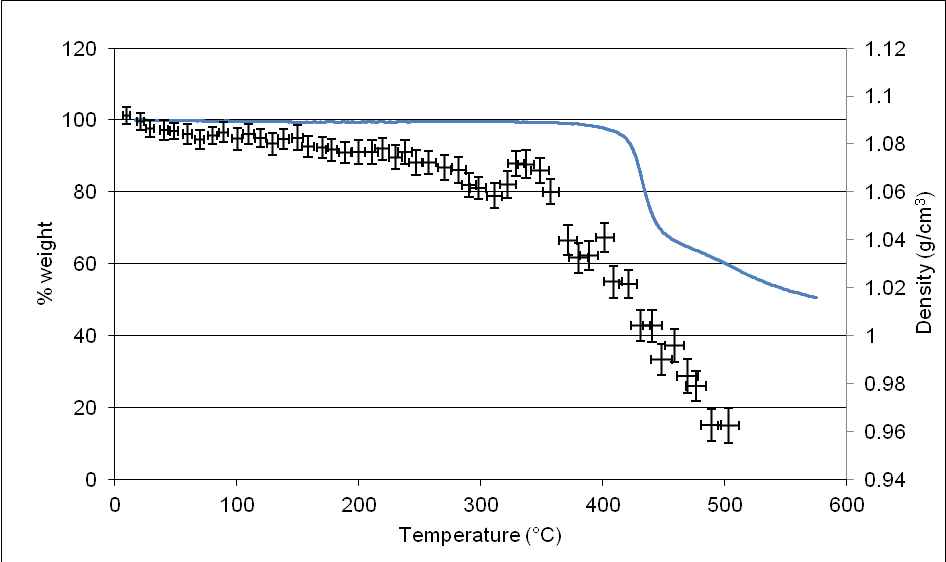


DMTA


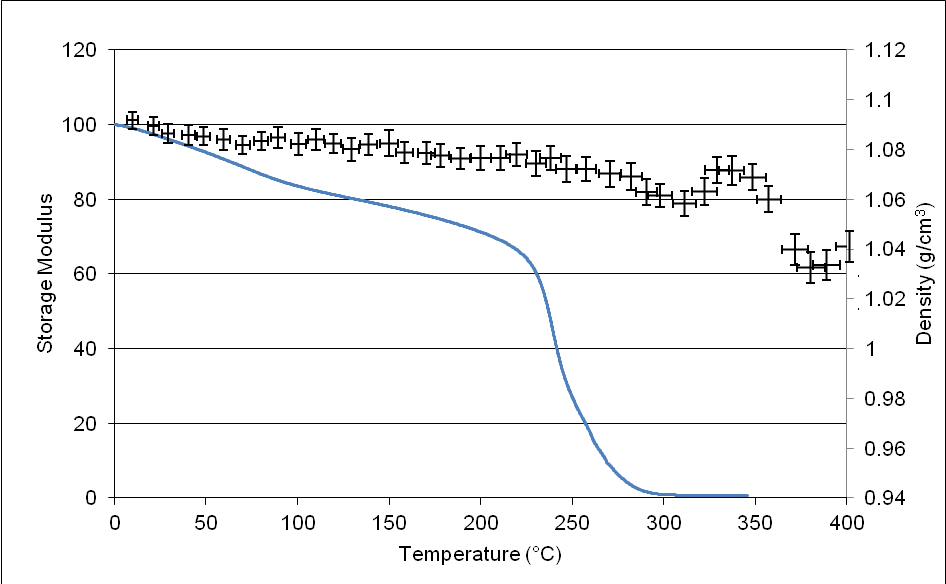


**1**_60_-**2**_40_

TGA


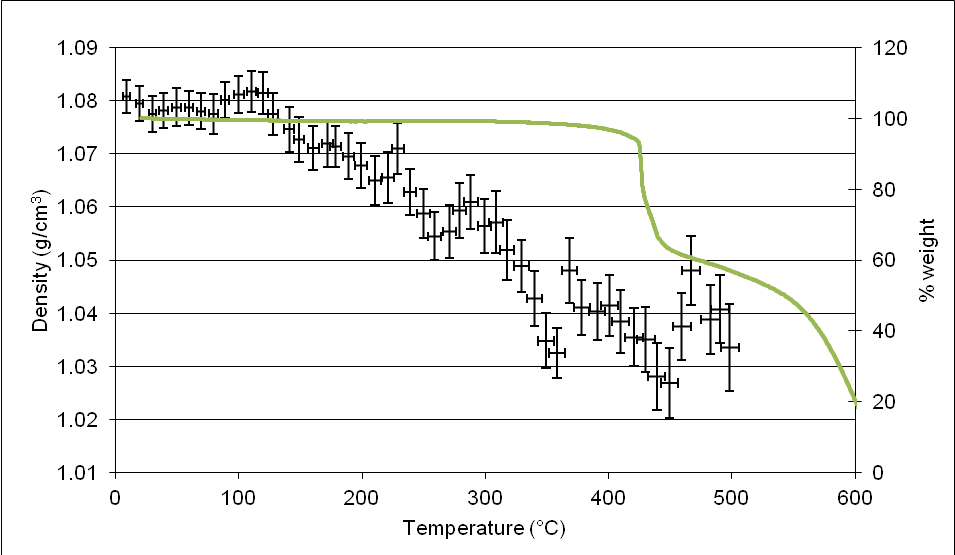


DMTA


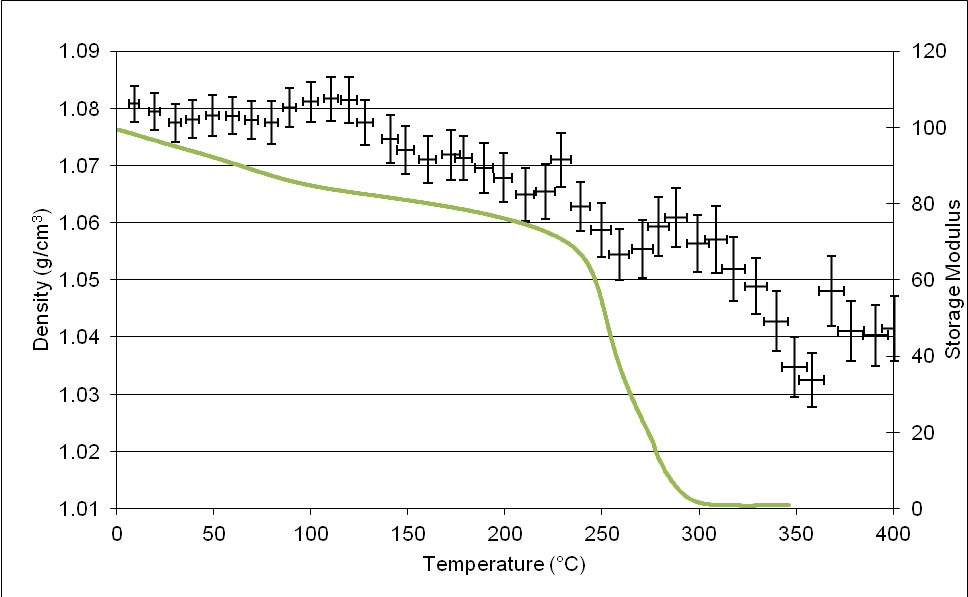


**1**_70_-**2**_30_

TGA


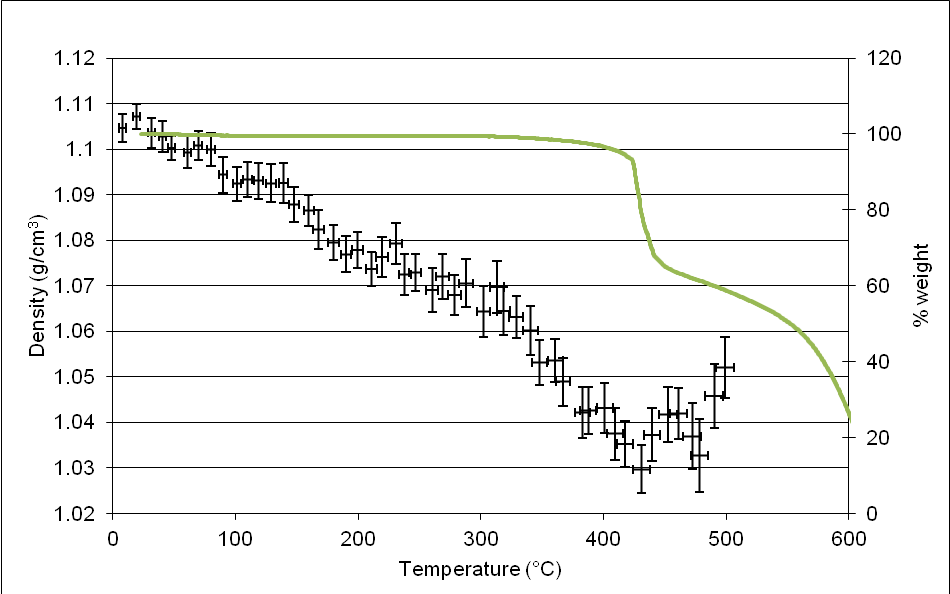


DMTA


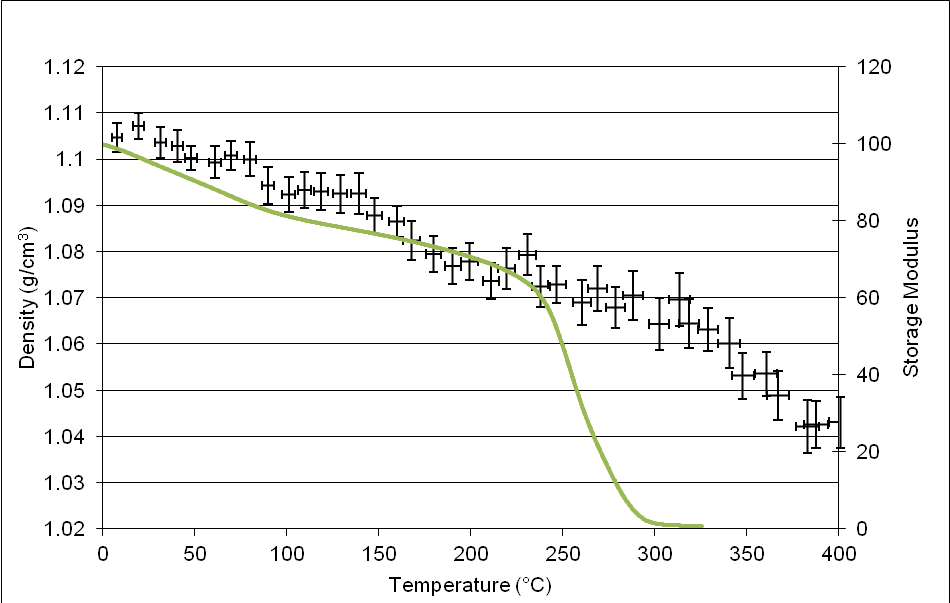


**1**_80_-**2**_20_

TGA


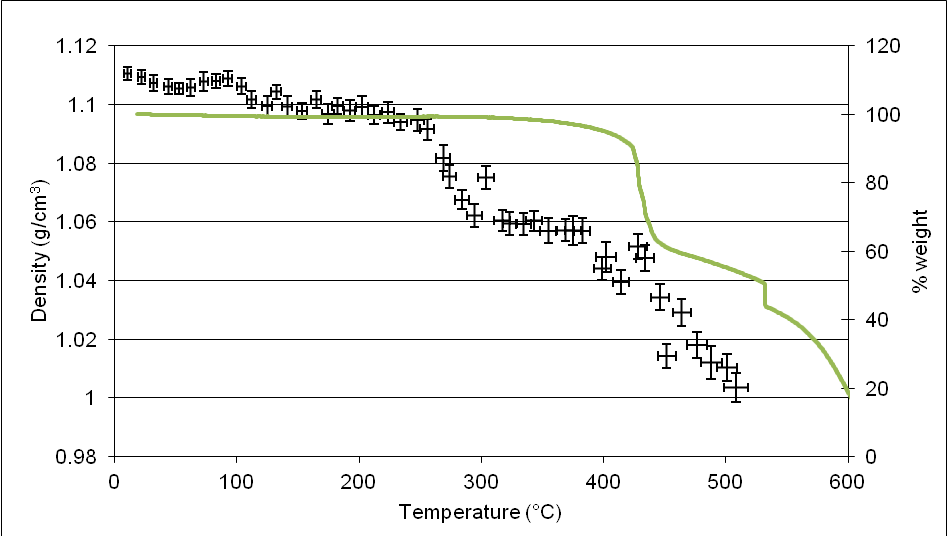


DMTA


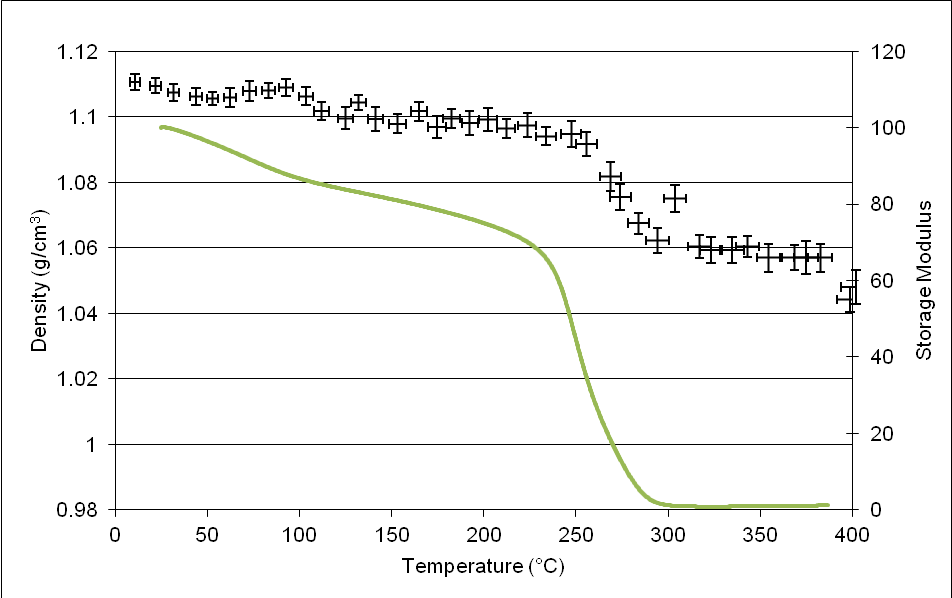


**1**_90_-**2**_10_

TGA


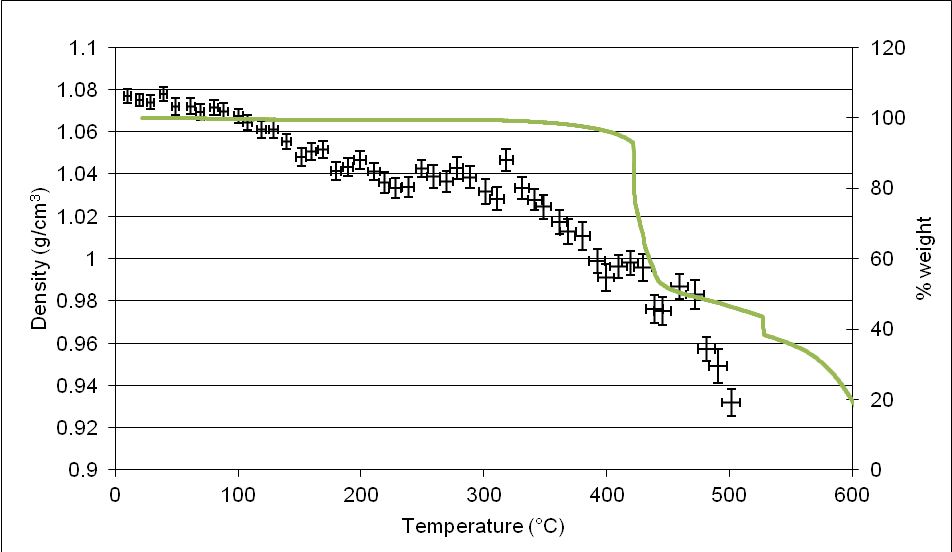


DMTA


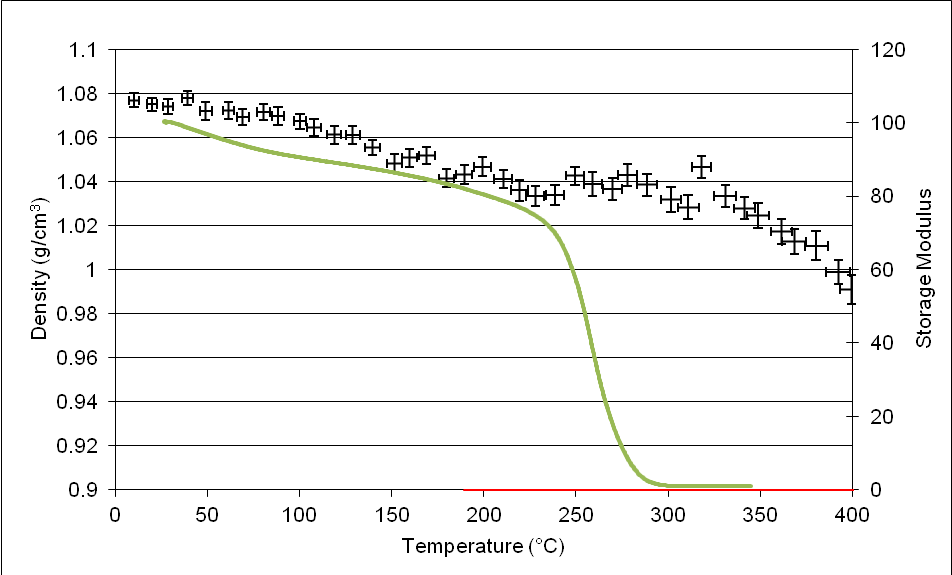

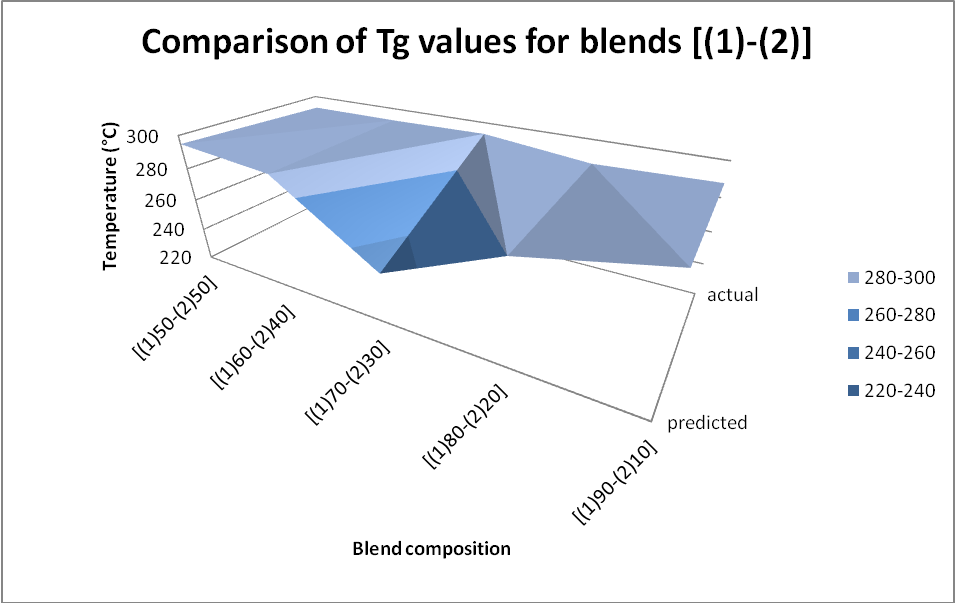

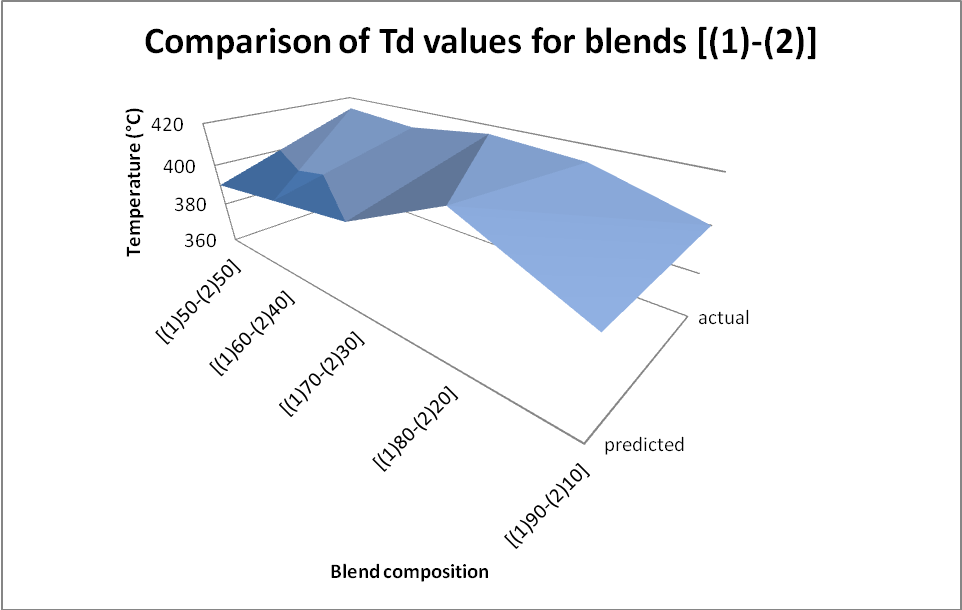


**3**_50_-**1**_50_

TGA


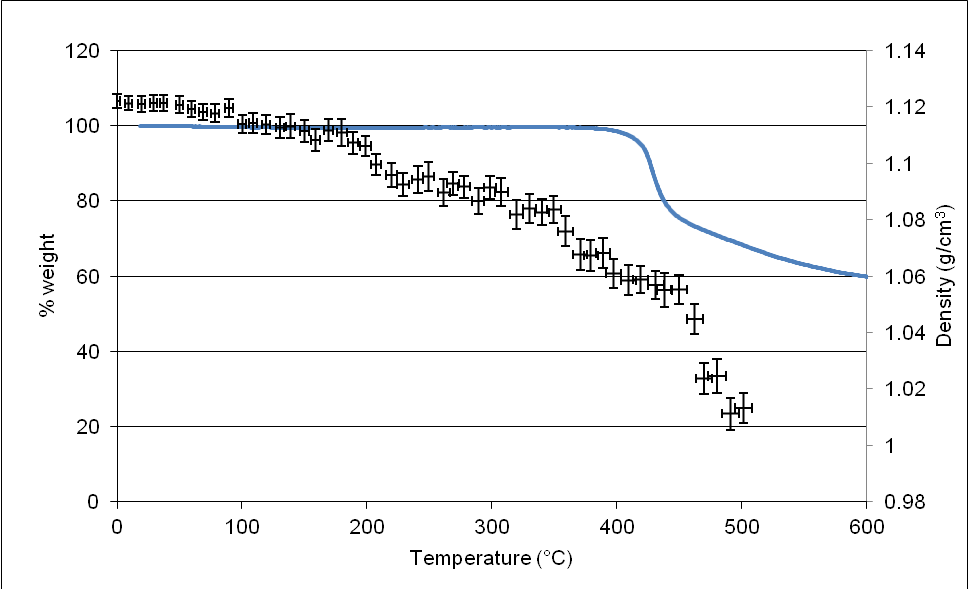


DMTA


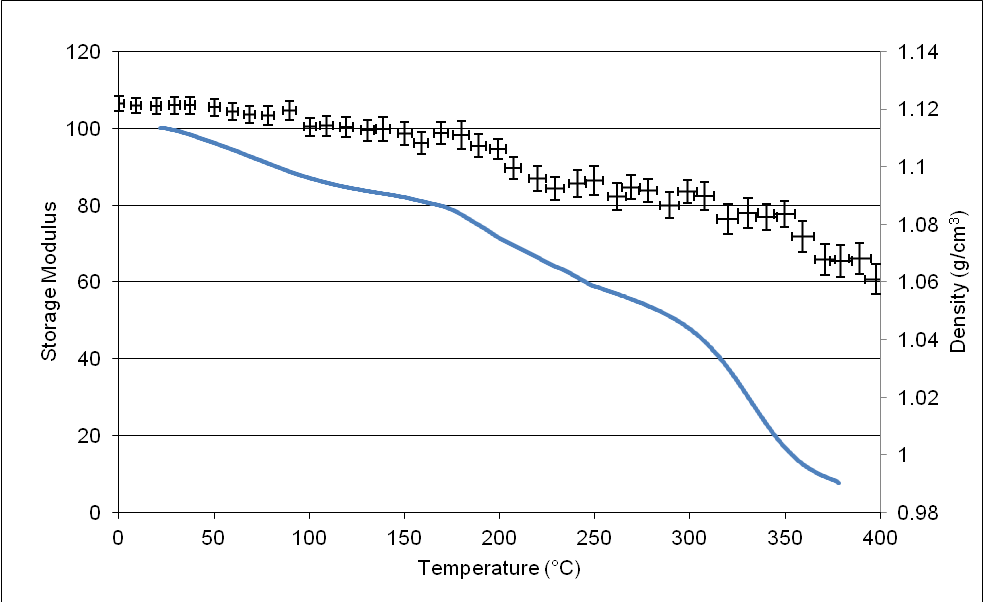


**3**_60_-**1**_40_

TGA


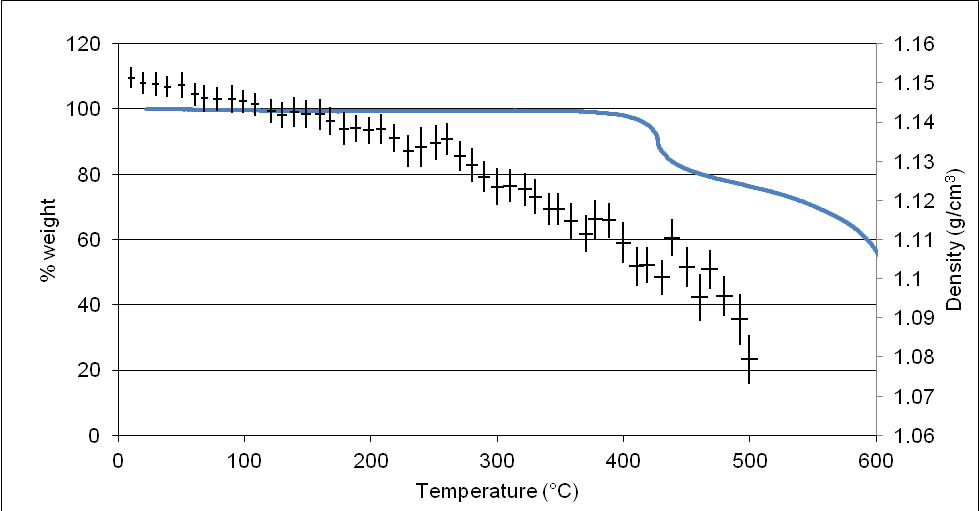


DMTA


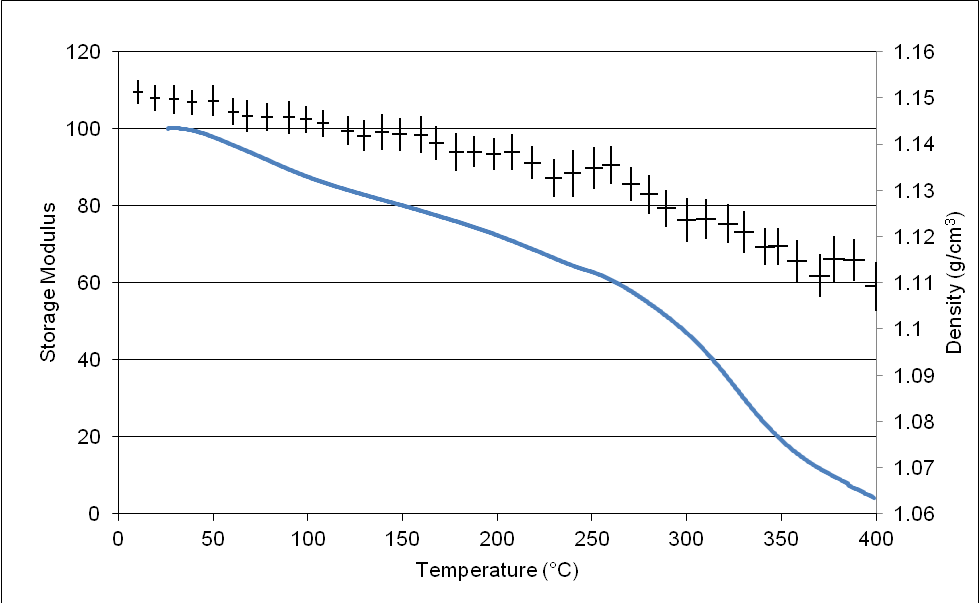


**3**_70_-**1**_30_

TGA


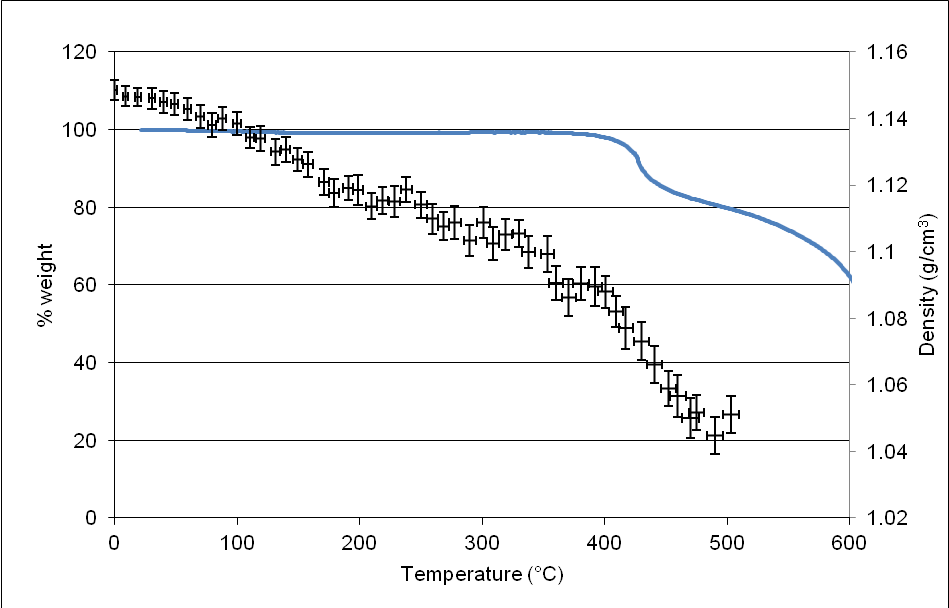


DMTA


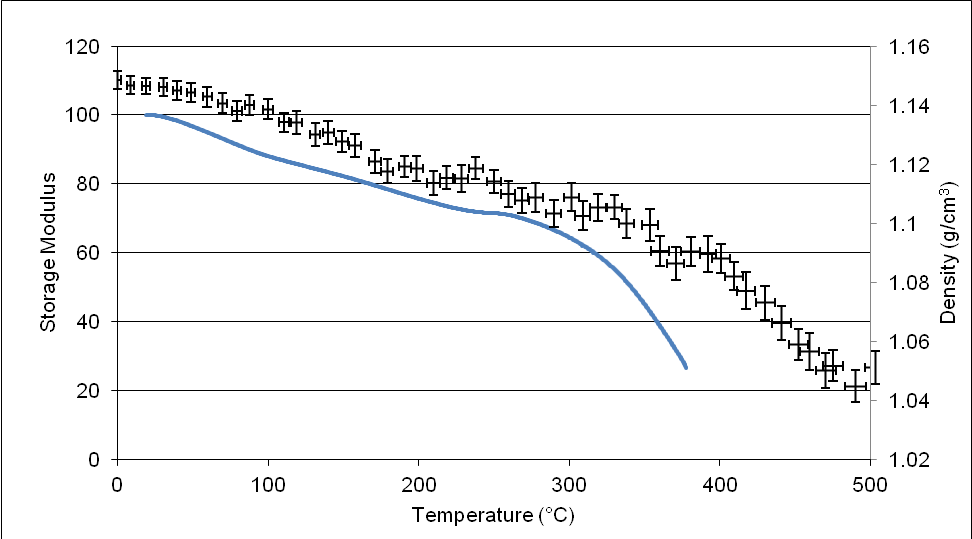


**3**_80_-**1**_20_

TGA


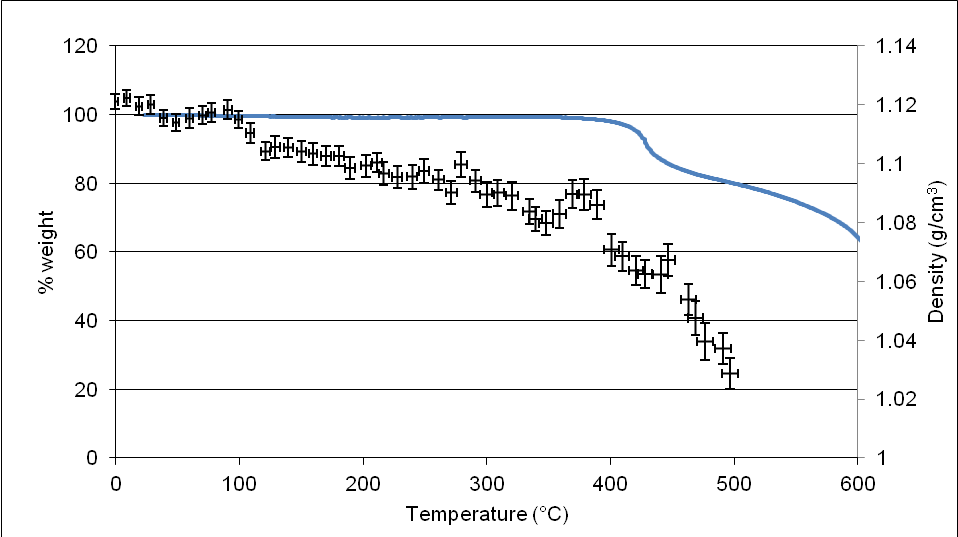


DMTA


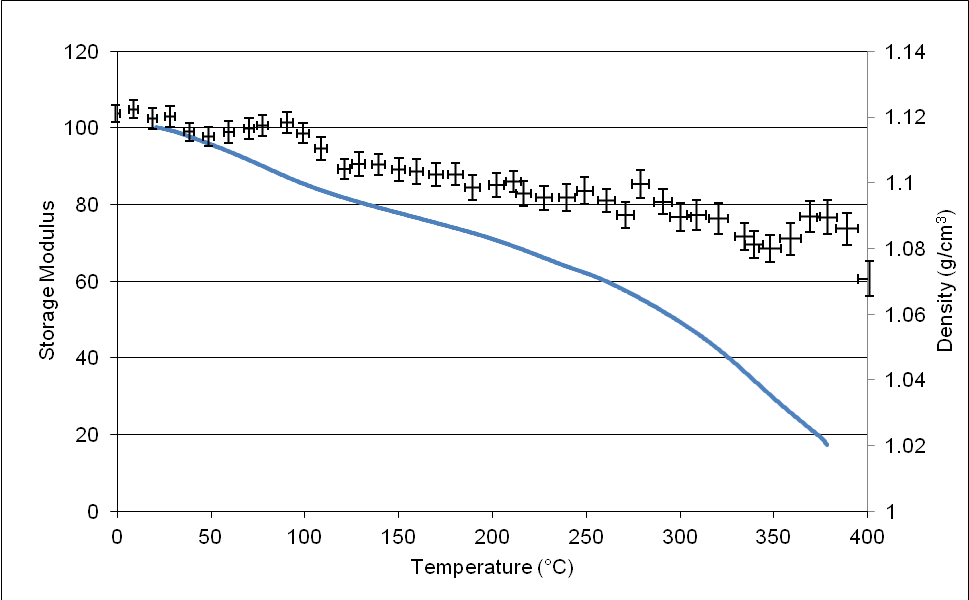


**3**_90_-**1**_10_

TGA


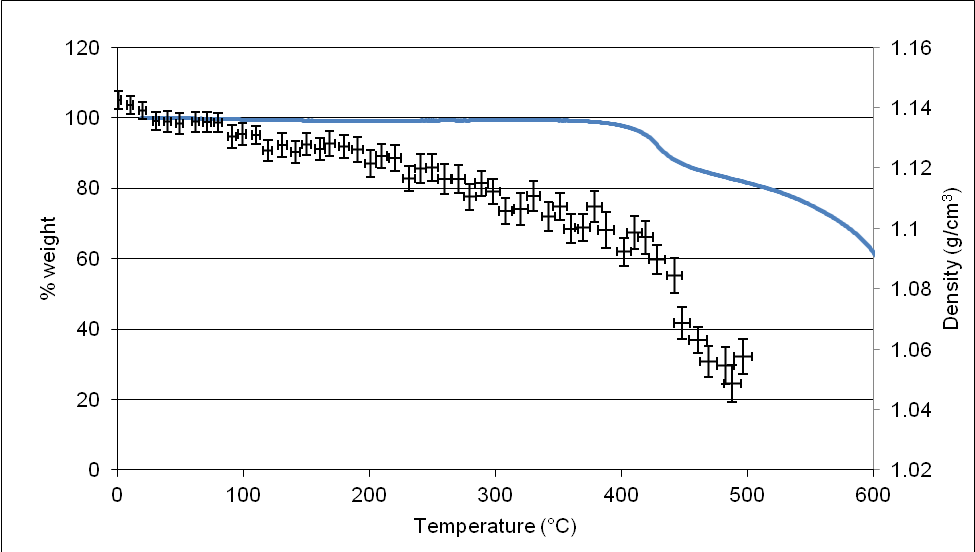


DMTA


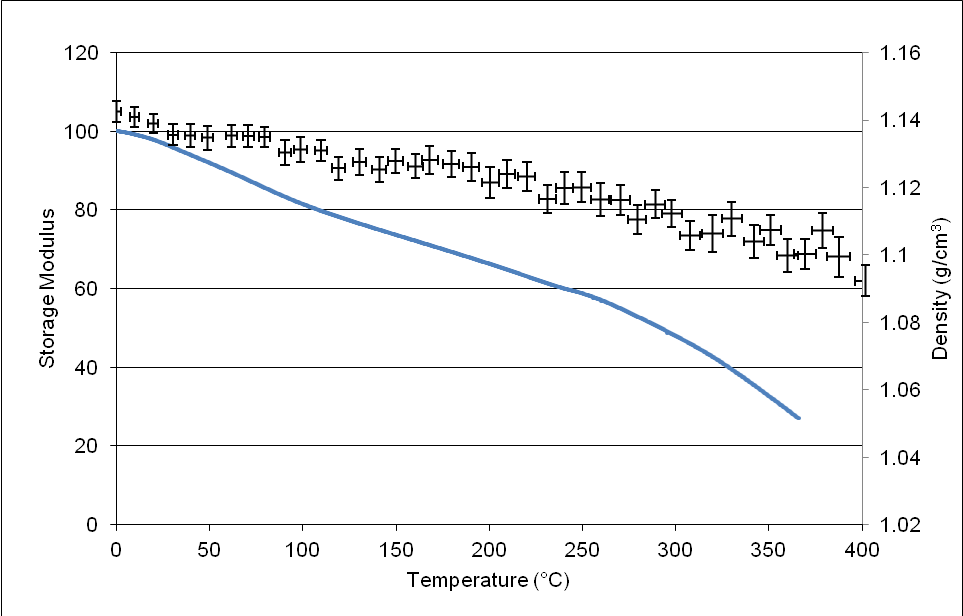

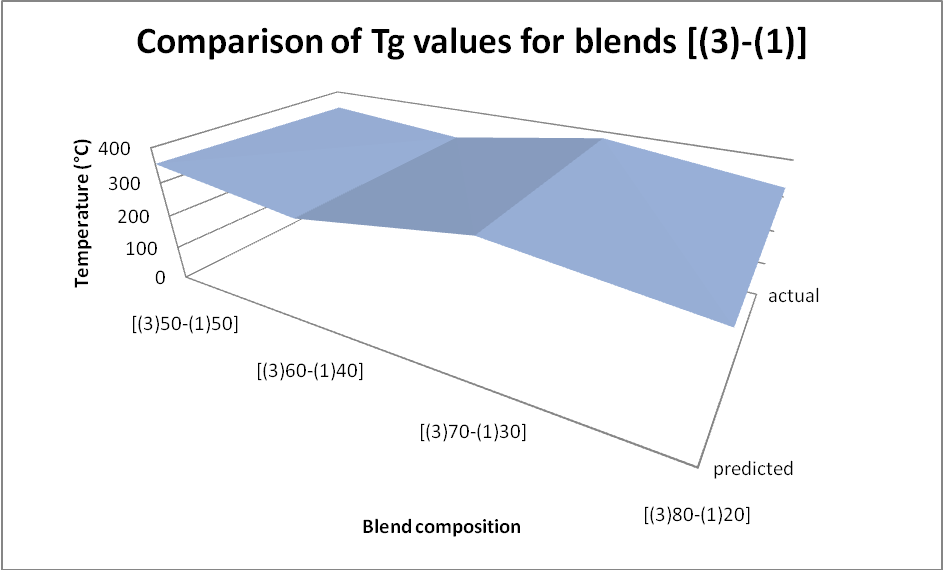

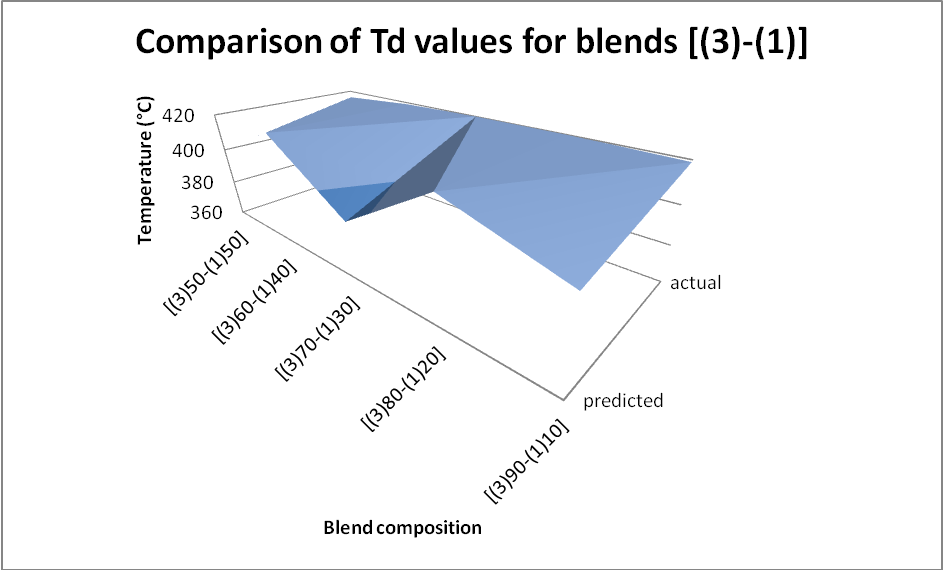


**3**_50_-**2**_50_

TGA


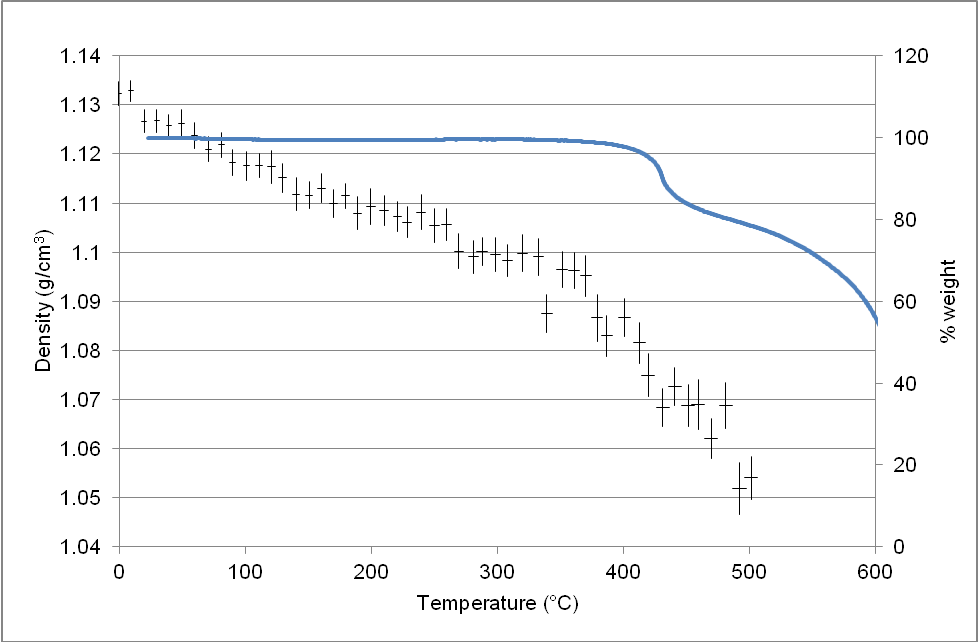


DMTA


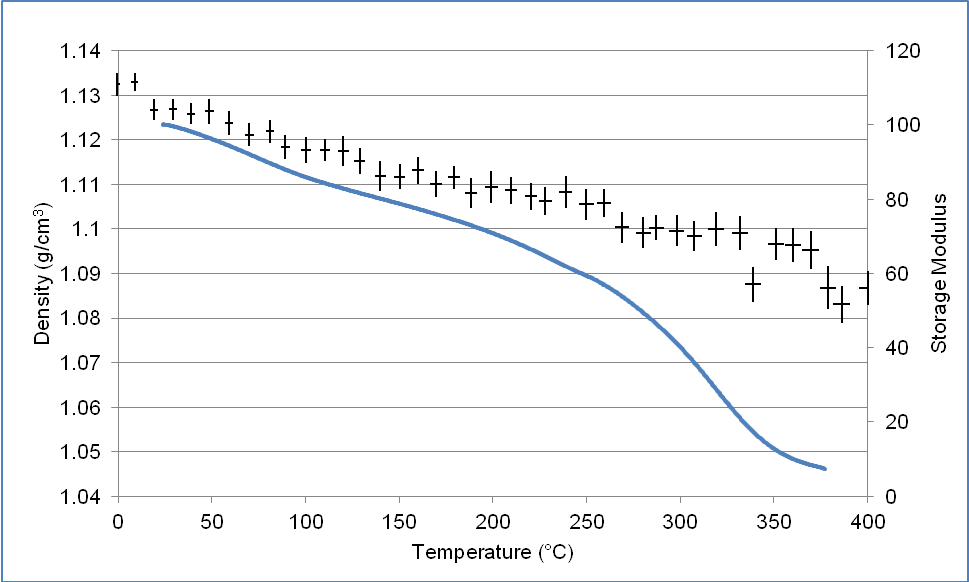


**3**_60_ -**2**_40_

TGA


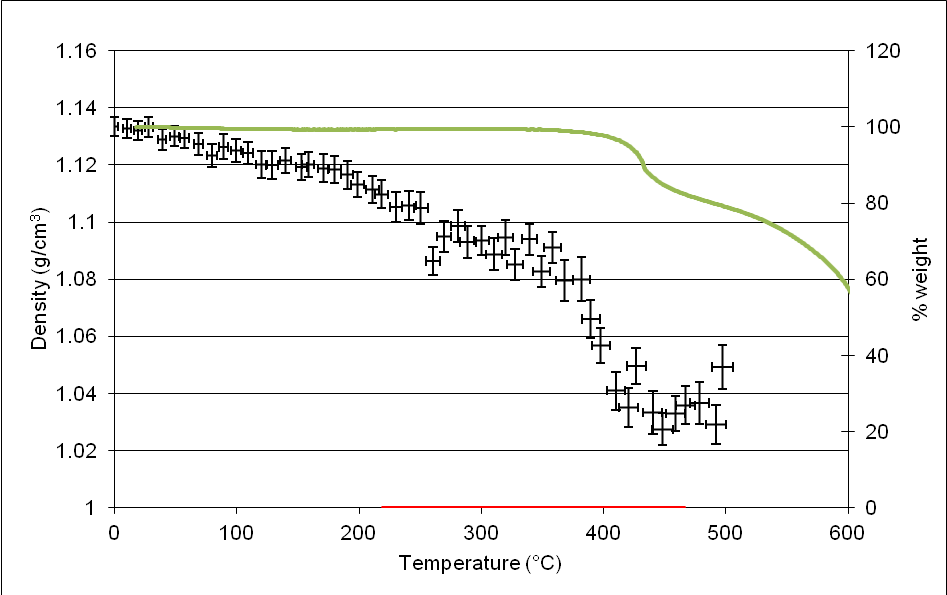


DMTA


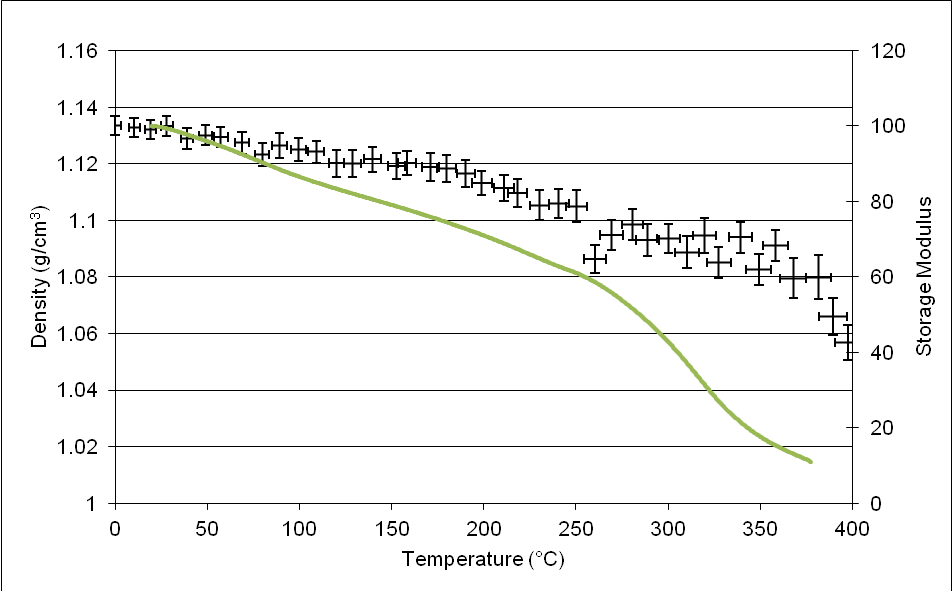


**3**_70_-**2**_30_

TGA


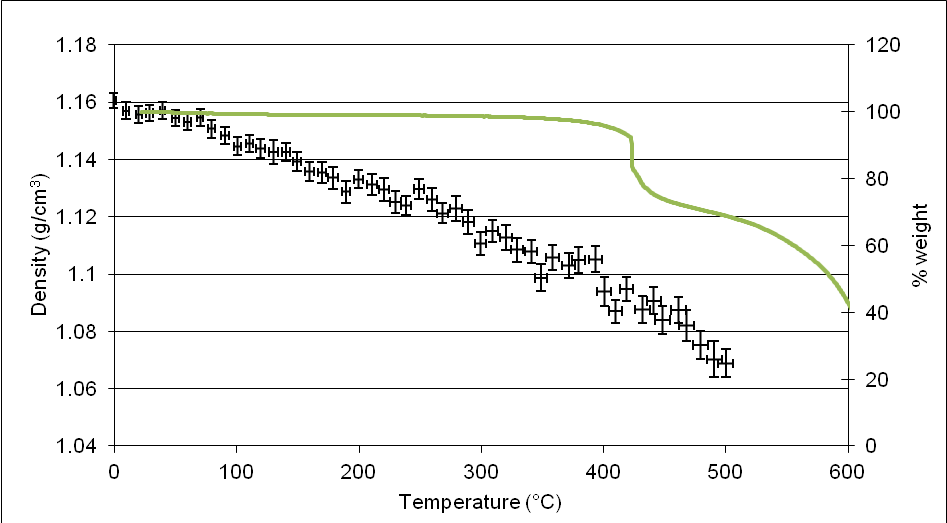


DMTA


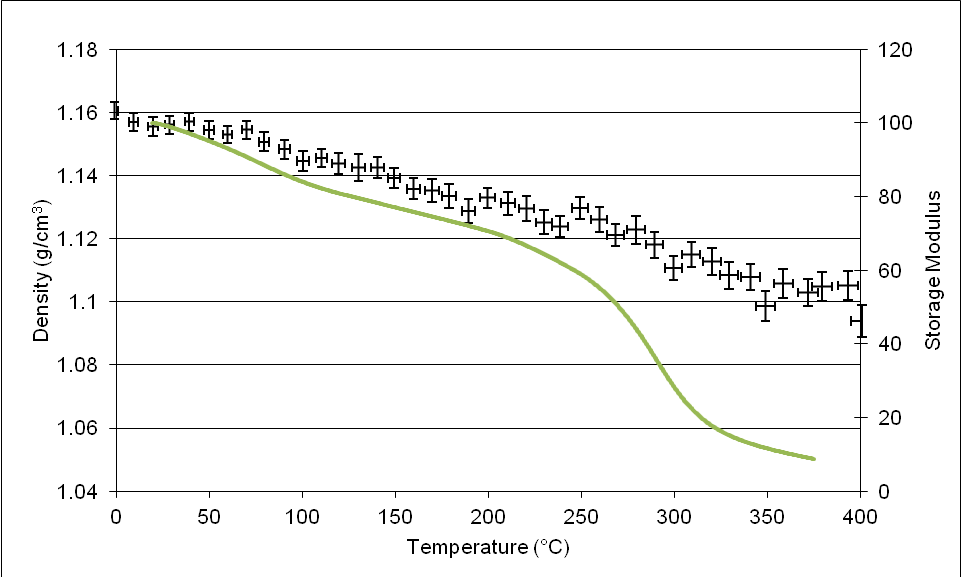


**3**_80_-**2**_20_

TGA


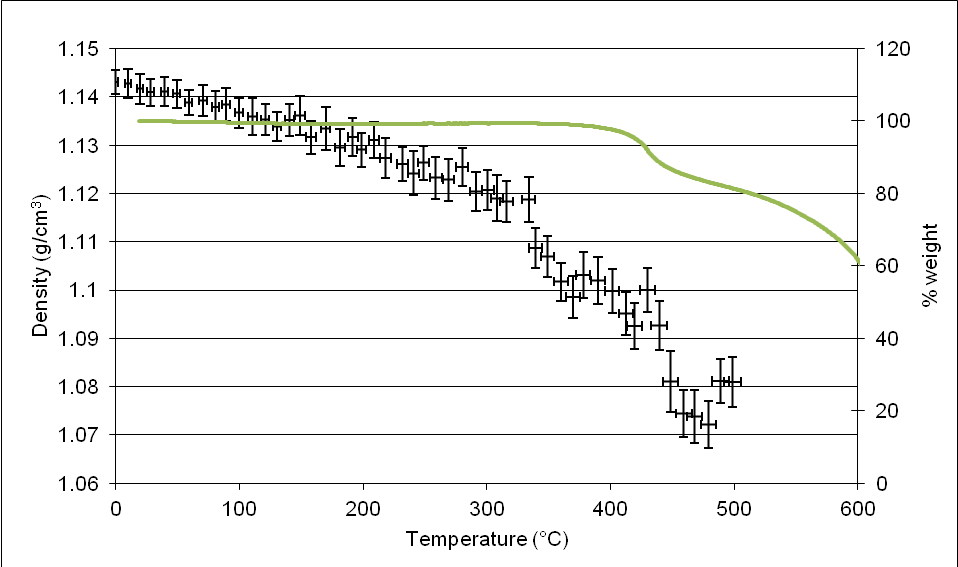


DMTA


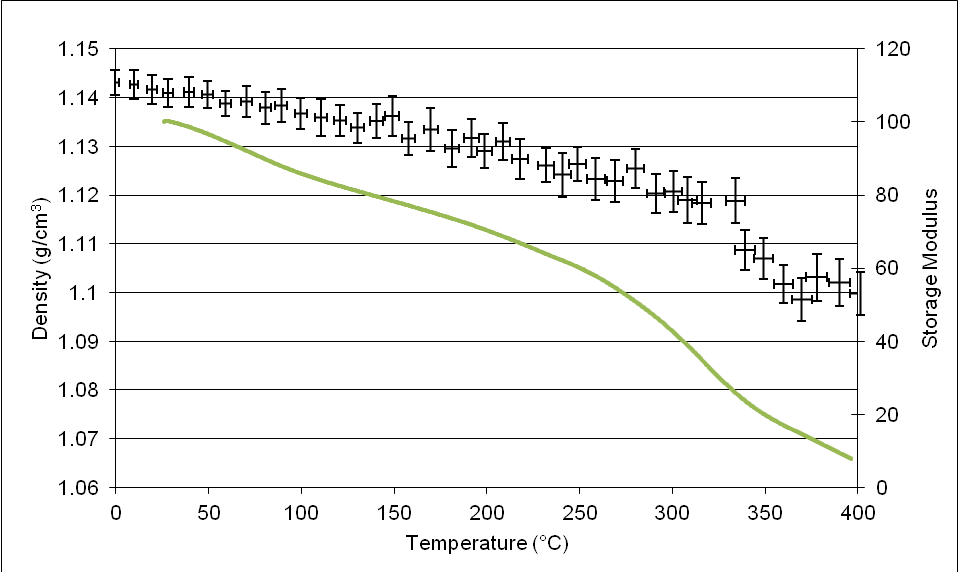


**3**_90_-**2**_10_

TGA


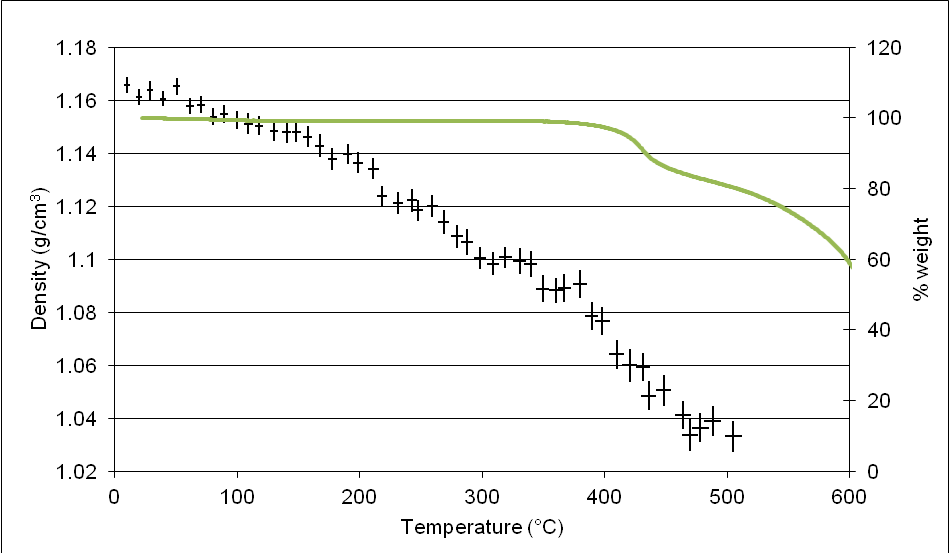


DMTA


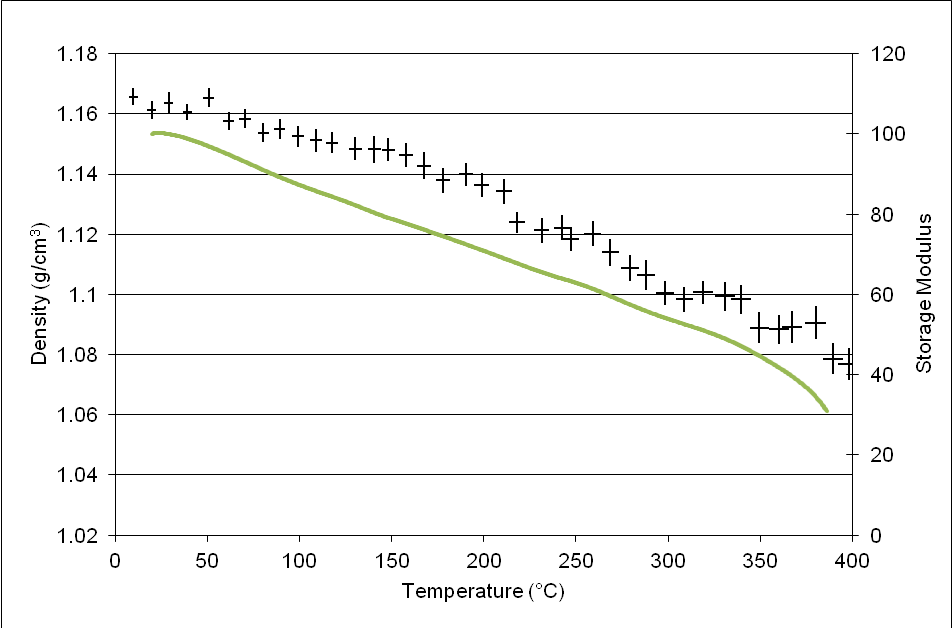

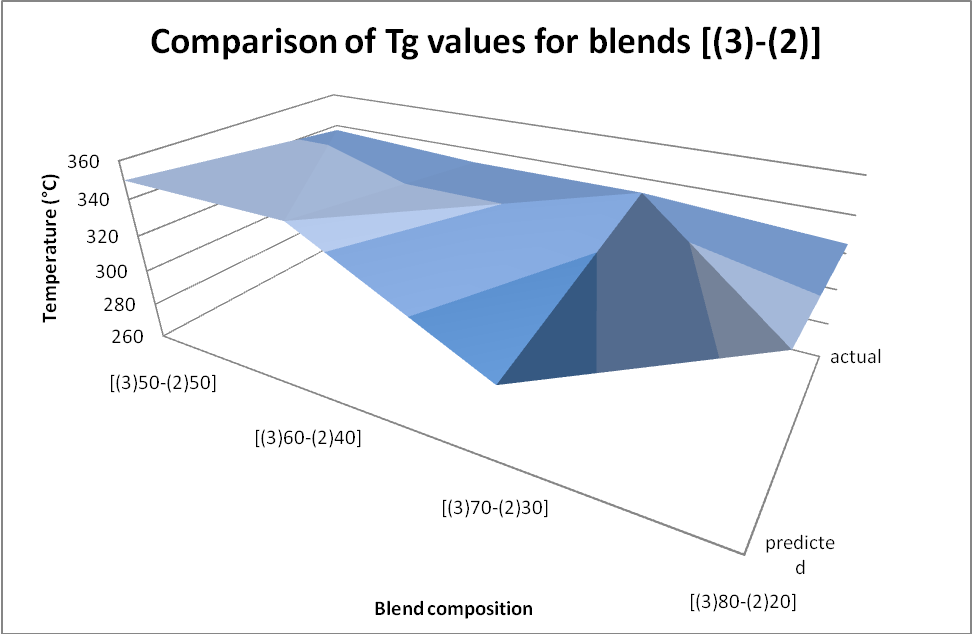

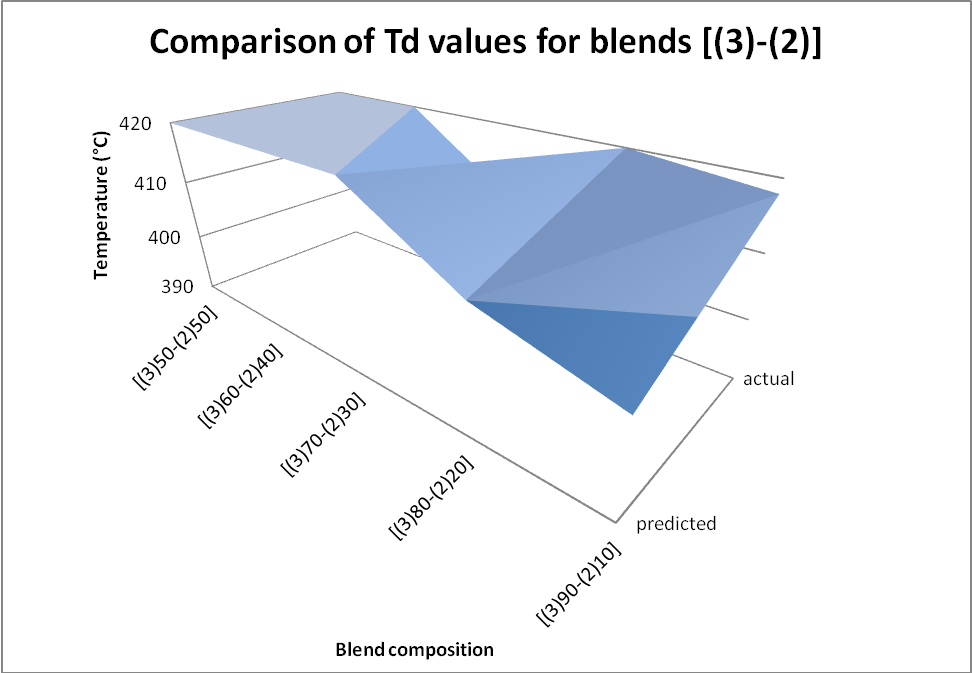

Supplement: Table S2 — A visual comparison of the experimental TGA and DMTA data plotted against the simulated data. (DOCX) [file pone.0044487.s002.docx]
